# Genomic composition and evolution of *Aedes aegypti* chromosomes revealed by the analysis of physically mapped supercontigs

**DOI:** 10.1186/1741-7007-12-27

**Published:** 2014-04-14

**Authors:** Vladimir A Timoshevskiy, Nicholas A Kinney, Becky S deBruyn, Chunhong Mao, Zhijian Tu, David W Severson, Igor V Sharakhov, Maria V Sharakhova

**Affiliations:** 1 Department of Entomology, Fralin Life Science Institute, Virginia Tech, Blacksburg, VA, USA; 2 Department of Genomics, Bioinformatics, and Computational Biology, Virginia Tech, Blacksburg, VA, USA; 3 Department of Biological Sciences, Eck Institute for Global Health, University of Notre Dame, Notre Dame, IN, USA; 4 Virginia Bioinformatics Institute, Virginia Tech, Blacksburg, VA, USA; 5 Department of Biochemistry and Fralin Life Science Institute, Virginia Tech, Blacksburg, VA, USA

**Keywords:** Physical mapping, Mosquito, Genome, Chromosome

## Abstract

**Background:**

An initial comparative genomic study of the malaria vector *Anopheles gambiae* and the yellow fever mosquito *Aedes aegypti* revealed striking differences in the genome assembly size and in the abundance of transposable elements between the two species. However, the chromosome arms homology between *An. gambiae* and *Ae. aegypti*, as well as the distribution of genes and repetitive elements in chromosomes of *Ae. aegypti*, remained largely unexplored because of the lack of a detailed physical genome map for the yellow fever mosquito.

**Results:**

Using a molecular landmark-guided fluorescent *in situ* hybridization approach, we mapped 624 Mb of the *Ae. aegypti* genome to mitotic chromosomes. We used this map to analyze the distribution of genes, tandem repeats and transposable elements along the chromosomes and to explore the patterns of chromosome homology and rearrangements between *Ae. aegypti* and *An. gambiae*. The study demonstrated that the q arm of the sex-determining chromosome 1 had the lowest gene content and the highest density of minisatellites. A comparative genomic analysis with *An. gambiae* determined that the previously proposed whole-arm synteny is not fully preserved; a number of pericentric inversions have occurred between the two species. The sex-determining chromosome 1 had a higher rate of genome rearrangements than observed in autosomes 2 and 3 of *Ae. aegypti*.

**Conclusions:**

The study developed a physical map of 45% of the *Ae. aegypti* genome and provided new insights into genomic composition and evolution of *Ae. aegypti* chromosomes. Our data suggest that minisatellites rather than transposable elements played a major role in rapid evolution of chromosome 1 in the *Aedes* lineage. The research tools and information generated by this study contribute to a more complete understanding of the genome organization and evolution in mosquitoes.

## Background

The genome of the major vector of arboviruses *Aedes aegypti*[1] was the second published mosquito genome after the genome of the malaria vector *Anopheles gambiae*[2]. The size of *Ae. aegypti* genome - 1,376 mega base pairs (Mb) - is the largest among other mosquito genomes sequenced so far. It is five times bigger than the 264 Mb genome of *An. gambiae*. The most striking difference between these two mosquito genomes is in the abundance of transposable elements (TEs). TEs cover approximately 50% of the *Ae. aegypti* genome [3]*versus* 16% in the malaria mosquito genome [2]. There are also differences in the karyotype structure between the two species. The mitotic chromosome complement of *Ae. aegypti* consists of three pairs of metacentric chromosomes [4]. The smallest, largest and intermediate chromosomes are numbered as 1, 2 and 3, respectively [5]. There are no sex chromosomes in *Ae. aegypti*; sex determination alleles have been linked to the smallest homomorphic autosome 1 [6]. By contrast, *An. gambiae* has two submetacentric autosomes and clearly distinguishable sex chromosomes: an acrocentric, half-heterochromatic X and a completely heterochromatic Y [7]. The *An. gambiae* genome is subdivided into two compartments - gene-rich euchromatin and gene-poor pericentromeric and intercalary heterochromatin [8]. TEs and satellites have been found to be the most abundant in the heterochromatic regions. Unlike in *An. gambiae*, TEs in the *Ae. aegypti* genome predominantly infiltrated introns of most of the protein-coding genes [1]. Although C-banding studies located heterochromatin in centromeres of all chromosomes and in the intercalary region on the q arm of the homomorphic sex-determining chromosome 1 of *Ae. aegypti*[9-11], molecular characteristics of the heterochromatic regions in this species remain unclear.

The availability of genome sequences for mosquitoes provides an opportunity to study the molecular structures of their chromosomes and the patterns of chromosome evolution. To facilitate this investigation, physical chromosome-based maps for various species of mosquitoes have been developed [12-15]. The most detailed polytene chromosome-based physical map was constructed for the malaria vector *An. gambiae*[2,15,16]. This map includes 2,000 bacterial artificial chromosome (BAC) clone markers and anchors 88% of the genome to the chromosomes. Lower resolution physical maps were also created for *An. funestus*[12] and *An. stephensi*[13]*.* Only about 31% of the *Ae. aegypti* genome was originally assigned to the chromosomes without specifying order and orientation [1] based on previous genetic mapping data [17,18]. A recent genetic mapping effort assigned 60% of the genome to 62 chromosome positions [19]. Although the physical maps of the mosquitoes are incomplete, they provide important insights into chromosomal evolution. A comparative study between *An. gambiae* and two other malaria mosquitoes, *An. funestus* and *An. stephensi*, demonstrated that chromosome arms have different rates of evolution associated with arm-specific genomic features [20,21]. The highest rate of evolution was detected in the sex chromosome X in association with an abundance of TEs and tandem repeats. A comparative cytogenomic study between *Aedes* and *Anopheles* shed light on the pattern of chromosomal evolution in mosquito lineages that diverged about 145 to 200 million years ago [22] and provided some clues about the evolution of homomorphic and heteromorphic sex chromosomes [1]. Coarse-scale chromosome comparison between *Ae. aegypti* and *An. gambiae* detected whole chromosome arm translocations between chromosomes 2 and 3 as well as the conserved gene orthology between the homomorphic sex-determining chromosome 1 of *Ae. aegypti* and both the X chromosome and autosome 2R arm of *An. gambiae*[1,23]. However, a fine-scale analysis of the chromosomal rearrangements in association with the genomic landscape has not been performed. The major limitation for this analysis is a lack of sufficient information about the position of genomic supercontigs on chromosomes of *Ae. aegypti.*

Physical mapping on polytene chromosomes of *Ae. aegypti* is difficult due to the low levels of polyteny, which result in poor quality chromosome preparations [24-26]. Furthermore, fluorescent *in situ* hybridization (FISH) experiments are complicated by the abundance of repetitive elements, which requires using unlabeled repetitive DNA fractions to block unspecific hybridization. The first physical map for *Ae. aegypti* was developed by FISH of 37 markers on mitotic chromosomes from the ATC-10 cell line [27]. This map was later integrated with the genetic linkage map [17] by direct placement of 27 DNA probes containing previously mapped genetic markers to chromosomes [28]. The map was distance-based, meaning that positions of the markers were determined by direct measurements of their locations on the chromosomes from the p terminus (FLpter). Recently, we introduced a band-based approach for the physical mapping of the *Ae. aegypti* genome to mitotic chromosomes [14,29]. Instead of previously used cell lines, which usually accumulate chromosome rearrangements [27,30], our method utilizes chromosomes from imaginal discs of fourth instar larvae. The positions of the probes are determined based on idiograms - schematic representations of the chromosome banding patterns. Idiograms have been constructed for chromosomes at early metaphase stained by YOYO-1 iodide. The three chromosomes of *Ae. aegypti* are subdivided into 23 regions and 94 subdivisions. Using FISH, 100 BAC clones were assigned to the specific bands on idiograms. These BAC clones contained previously mapped genetic markers as determined by PCR [31]. All BAC clones were additionally ordered within each band by multicolor FISH [14]. In addition to 100 genetic markers and 183 Mb of genomic sequences, a marker linked with sex determination [18] and 12 quantitative trait loci (QTL) associated with pathogen transmission [32-36] were also anchored to the chromosomes. However, the available physical map covered only 13.3% of the genome, and it required further improvement.

In this study, we constructed a more detailed physical map of the *Ae. aegypti* genome. Together with our previous mapping [14], a total of 624 Mb equal to approximately 45% of the *Ae. aegypti* genome were assigned to chromosome bands on idiograms. Our study revealed differences among chromosome arms in the composition of genomic features, such as genes, tandem repeats and TEs. We demonstrated that the sex-determining chromosome 1 has a significantly higher coverage of minisatellites than observed with chromosomes 2 and 3. We also investigated chromosome rearrangements between *Ae. aegypti* and *An. gambiae.* Our data demonstrated that previously proposed whole-arm synteny [1,23] is not fully preserved; a number of pericentric inversions have occurred between culicines and anophelines. Mapping 1:1 orthologs and microsynteny blocks common to *Ae. aegypti* and *An. gambiae* suggests a higher rate of gene reshuffling in the sex-determining chromosome of *Ae. aegypti* compared with chromosomes 2 and 3. Further development of a high-resolution physical map of the *Ae. aegypti* genome will lead to a significant improvement in the genome assembly and will guide future efforts to study genome organization and chromosome evolution in mosquitoes.

## Results

### A physical map of the *Ae. aegypti* genome

**Figure 1:**
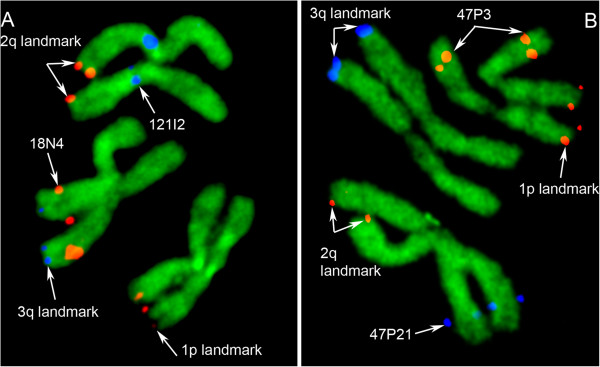
**A landmark-guided fluorescent *in situ *hybridization mapping of *Ae. aegypti *chromosomes.** The positions of bacterial artificial chromosome (BAC) clones used as chromosome arm landmarks and locations of BAC clones of interest on chromosomes **(A)** 2 and 3 and **(B)** 1 and 2 are indicated by arrows.

**Figure 2:**
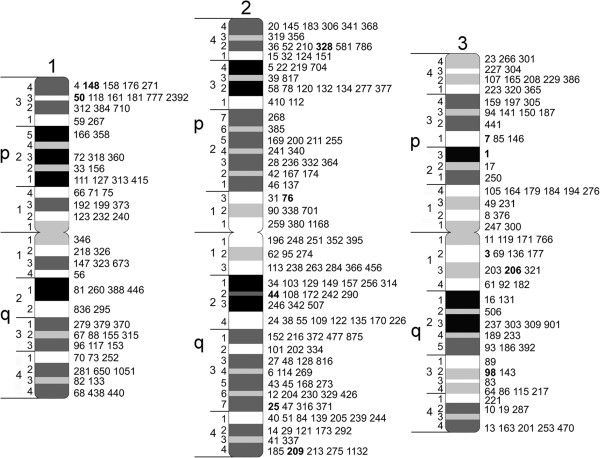
**A physical map of the *Ae. aegypti *genome.** Chromosome divisions and subdivisions are indicated on the left side of the idiograms. The positions of supercontigs are shown on the right side of the idiograms. Supercontig identities are indicated by the last two to four digits. Supercontigs in conflict with previous genetic mapping/genome assemblies are in bold.

Our study developed a physical map of 45% of the *Ae. aegypti* genome using FISH with mitotic chromosomes from imaginal discs of fourth instar larvae (Figure 1). The physical mapping was conducted based on previously developed idiograms for early metaphase chromosomes stained with YOYO-1 iodide [14]. For better accuracy and efficiency of mapping, we used a molecular landmark-guided mapping approach, which has been previously introduced [37]. Two-color hybridization of BAC clones was performed in the presence of three landmark probes with known locations in each of the three chromosomes of *Ae. aegypti* (Figure 1). For landmarks, we utilized BAC clones that produced strong unique signals in telomeric regions of the chromosomes. Using this approach, we hybridized 400 BAC clones from the *Ae. aegypti* NDL BAC library [31] to the chromosomes. The positions of the BAC clones within the largest genomic supercontigs were determined by a PCR-based library screening approach. From the 400 BAC clones isolated, 368 clones were successfully hybridized and mapped to the chromosomes. Together with the previously mapped 100 genomic supercontigs [14], a total of 294 supercontigs or 624 Mb of the *Ae. aegypti* genome were assigned to 91 of 94 total bands on chromosomes (Figure 2, Table 1, Additional file 1: Table S1). None of the BAC clones hybridized to region 1p24, pericentromeric region 1p11 and 3q43, region next to the telomere. Almost half of all supercontigs were mapped to the biggest chromosome 2. About 45% of *Ae. aegypti* genomic sequences and 47% (7,300 out of 15,500) of the protein-coding genes were placed in the specific locations of the chromosomes. The genomic supercontigs were assigned to the chromosome bands without ordering and orientation within the band. The order of the genomic supercontigs on our map was highly consistent with a recently published genetic linkage map [19], with Spearman’s rank correlation coefficients equal to 0.77, 0.83 and 0.65 (*P* <0.05) for chromosomes 1, 2 and 3, respectively (Additional file 2: Figure S1).

**Table 1:** **Results of a physical mapping of the *Ae. aegypti *** **genome**

**Mapping parameters**	**Number (percentage)**
Number of BAC clone	500
FISH results suitable for mapping	468 (94%)
Assigned supercontigs	294
Assigned to chromosome 1	70 (23%)
Assigned to chromosome 2	142 (48%)
Assigned to chromosome 3	82 (29%)
Mapped portion of the genome	624 Mb (45%)

BAC, bacterial artificial chromosome; FISH, fluorescent *in situ* hybridization.

Our physical mapping detected 29 cases of potential misassembly of genomic supercontigs. In these cases, two or more BAC clones identified in the same supercontig hybridized to very different chromosomal locations. Of these, 12 supercontigs (1.2, 1.30, 1.54, 1.74, 1.80, 1.91, 1.99, 1.154, 1.243, 1.286, 1.288 and 1.302) contained only two BAC clones hybridized to the different chromosome bands, and we indicated their positions on the chromosomes as unknown (Additional file 1: Table S1). Supercontigs 1.11, 1.12, 1.20, 1.31 and 1.48, which contained more than two mapped BAC clones, were assigned to the chromosomes based on the majorities of FISH results. The other 12 supercontigs (1.1, 1.3, 1.7, 1.25, 1.44, 1.50, 1.76, 1.98, 1.148, 1.206, 1.209 and 1.328) were previously assigned to chromosomes [14], and we also kept them on the map because their chromosome positions have been confirmed by genetic mapping [18]. These supercontigs are indicated in bold on the chromosome map (Figure 2). In 84 of 119 cases (73%), when we mapped two or more BAC clones from the same supercontig to the chromosomes, the mapping results were consistent with the genome data, indicating a proper assembly of these supercontigs.

### Genomic composition of *Ae. aegypti* chromosomes

**Figure 3:**
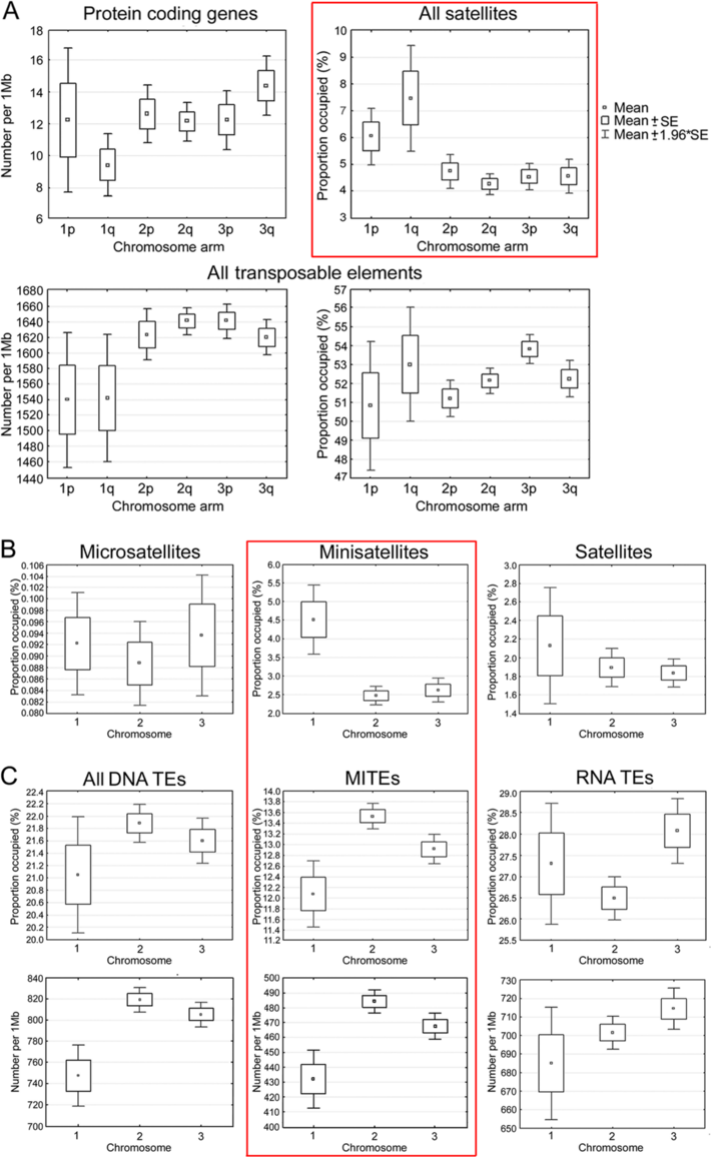
**Distribution of molecular features in *Ae. aegypti *chromosome. (A)** Genes, satellites and TEs in chromosome arms; **(B)** satellites and transposable elements in chromosomes; **(C)** transposable elements in chromosomes. Densities or coverage of the molecular features per supercontig are indicated on the Y axis; chromosome arms or chromosomes are shown on the X axis. The diagrams with the most striking differences in molecular features between chromosome and chromosome arms are in red boxes. Mb: mega base pairs; MITEs: miniature inverted-repeat transposable element; SE: standard error; TEs: transposable elements.

We used the physical map developed by this study to compare genomic features, such as genes, satellites and TEs, in individual chromosomes of *Ae. aegypti* (Figure 3). The average protein-coding gene densities per Mb were 10.9 in chromosome 1, 12.3 in chromosome 2 and 13.4 in chromosome 3. Although statistical analysis of protein-coding gene numbers revealed no significant differences among the chromosomes (*P* = 0.12), the sex-determining chromosome 1 was slightly but significantly different from chromosome 3, if mild criterion for pair comparisons (least significant difference test *P* = 0.04) was used. Among chromosome arms, the lowest gene density of 9.42 per Mb was found in the q arm of the homomorphic sex-determining chromosome (Figure 3A). Non-conservative pair comparison showed that the gene densities in chromosome 1 q arm were significantly different from gene densities in 2p (*P* = 0.048) and 3q (*P* = 0.004). In contrast to genes, tandem repeats were significantly more abundant in chromosome 1. Chromosome 1 had 6.73% of tandem repeats compared with 4.46% in chromosomes 2 (*P* <10^−7^) and 4.55% in chromosome 3 (*P* <10^−7^) (Figure 3). The abundance of minisatellites (7 to 99 base-pair (bp)-long repeat units) in chromosome 1 provided the major contribution to these differences (Figure 3B). Coverage of microsatellites (2 to 6 bp-long repeat units) and large satellites (>100 bp-long repeat units) was not significantly different among the chromosomes. Our analysis also revealed significantly higher overall density of all satellites on the q arm (Figure 3A) of chromosome 1 (8.80%, *P* <10^−7^).

The overall coverage of TEs was almost equal among chromosomes: 51.9% on chromosome 1, 51.8% on chromosome 2 and 53.0% on chromosome 3 (*P* = 0.25) (Figure 3). The analysis of various types of TEs revealed differences in classes distributed among the chromosomes (Figure 3C). For example, chromosome 1 had a lower density of Class II, DNA-mediated transposons (747.2 per Mb) than chromosomes 2 (819.1 per Mb, *P* <10^−7^) and 3 (805.1 per Mb, *P* = 1.3×10^−4^). All chromosomes differed in the abundance (coverage and counts) of miniature inverted-repeat TEs (MITEs). This class of short, 500 bp-long TEs belongs to DNA-mediated TEs. MITEs were most abundant in chromosome 2 (13.5%, 484.3 per Mb) as compared with chromosomes 1 (*P* <10^−4^) and 3 (*P* = 1.3×10^−2^). Chromosome 3 had a higher coverage of Class I, RNA-mediated TEs than chromosome 2 (26.5% versus 28.1%, *P* = 2×10^−2^). Interestingly, the overall densities of TEs were slightly lower in sex-determining chromosome 1 (1,540 per Mb) than in chromosomes 2 (1,634 per Mb; *P* = 2.2×10^−5^) and 3 (1,630 per Mb; *P* = 2.73×10^−4^). This fact can be explained by the differences in sizes between different classes of TEs and preferable location of the TEs in gene introns that makes them abundant in the gene-rich environment on *Ae. aegypti* chromosomes 2 and 3.

**Figure 4:**
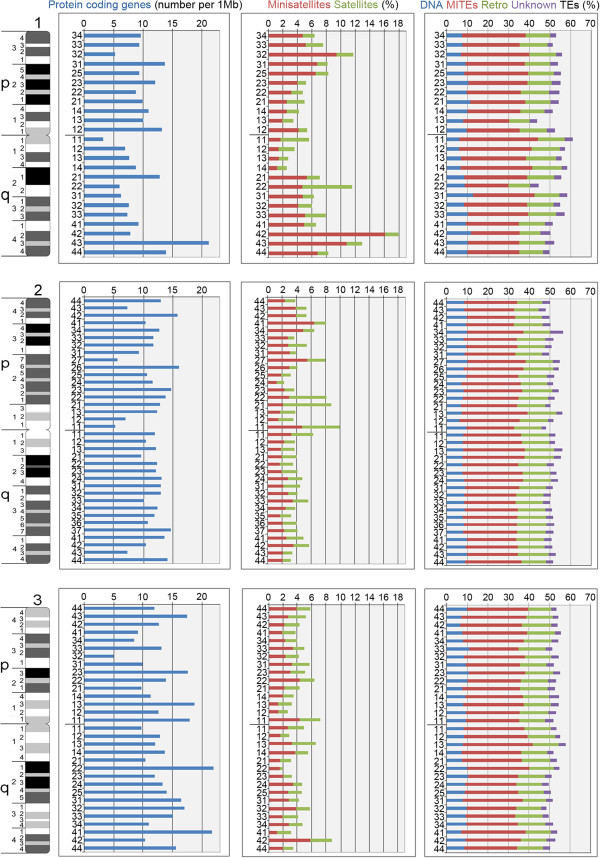
**Genome landscape of genes, satellites and transposable elements along chromosomes of *Ae. aegypti*.** Images on the left side of the figure represent idiograms for chromosomes 1, 2 and 3. The average amounts of different molecular features per chromosome band are shown on the charts by different colors. Mb: mega base pairs; MITEs -miniature inverted-repeat transposable element; TEs: transposable elements.

In addition to the interchromosome comparison, we also analyzed the landscape of molecular features along the three chromosomes of *Ae. aegypti* (Figure 4). Despite the fact that the physical map constructed here covers only approximately 45% of the *Ae. aegypti* genome, our study demonstrated that the densities of genes in areas around the centromeres of chromosomes 1 and 2 were lower than the average in the chromosomes. By contrast, the coverage of satellites was significantly higher around the centromeres in regions 1q11, 2p11 and 3p11 (*P* <10^-5^). A dearth of genes (5.9 per Mb, *P* <10^-5^) and abundance of satellites (11.54%, *P* <10^-5^) were also found in region 1q22. The location of the ribosomal genes in this region has been shown previously [29]. Moreover, scaffold 1.836, which contains the rDNA region, had even higher satellite content (24.1%), whereas the average satellite content in all chromosomes was equal to 5.02%. Thus, pericentromeric areas and region 1q22 have molecular characteristics of heterochromatin [38]. An additional peak of satellites was found in the subtelomeric region 1q42 to 1q43 (17.97% and 12.96%). TEs did not demonstrate significant differences in their distribution along the chromosomes.

### Chromosome rearrangements between *Ae. aegypti* and *An. gambiae*

**Figure 5:**
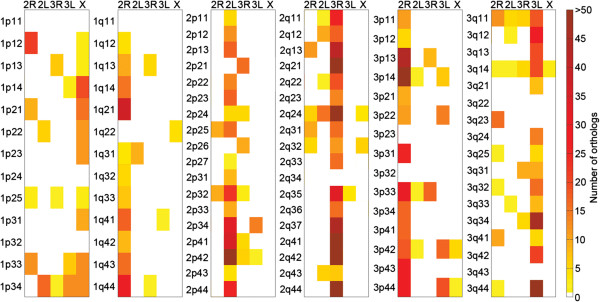
**Chromosome map of orthologous genes between *Ae. aegypti *and *An. gambiae*.** The intensities of the color indicate differences in numbers of orthologs in each region (scale in the right column). The Y axis shows subdivisions of the *Ae. aegypti* chromosomes. The X axis represents chromosome arms of *An. gambiae*.

The synteny between the chromosome regions in *Ae. aegypti* and *An. gambiae* was investigated using the physical map developed in this study. This investigation of synteny excluded 40 misassembled supercontigs identified by genetic linkage mapping [19]. A total of 2,335 out of 5,265 *Ae. aegypti-An. gambiae* orthologs were mapped to the 254 properly assembled supercontigs of the *Ae. aegypti* genome (Figure 5). Almost each subregion of *Ae. aegypti* had orthologs from more than one *An. gambiae* chromosome. Nine from 94 subregions contained zero orthologs due to the low number of mapped supercontigs. The number of orthologs per region varied from 1 to 75. The number of shared orthologs between *Ae. aegypti* and *An. gambiae* in each region is shown by the color intensities in Figure 5. A higher number of shared orthologs per region were found in the more densely mapped chromosome 2. In general, the results recapitulated the *Ae. aegypti* and *An. gambiae* chromosome arm synteny identified previously [1,23]. However, the physical map developed here provides a more detailed view of synteny at subregion resolution. Our data support the previous finding that the 1p and 1q arms of *Ae. aegypti* mostly contain the genomic material from the X and 2R chromosome arms of *An. gambiae*, respectively, supporting the idea of large translocation between the X and part of the 2R chromosome arm. In addition, we found a clear correspondence between the X chromosome of *An. gambiae* and region 1q22, known to contain the ribosomal locus in *Ae. aegypti*. We also found a synteny block between the 2R arm of *An. gambiae* and the 1p arm of *Ae. aegypti* in subregion 1p12. These results indicate the possibility of pericentric inversions during the evolution of the sex-determining chromosome in mosquitoes. The presence of additional large blocks from *An. gambiae* arms 2L and 3R on chromosome 1 of *Ae. aegypti* suggests even more complex evolution of this chromosome in *Ae. aegypti*. Observations of the synteny between chromosomes 2 and 3 in *Ae. aegypti* and *An. gambiae* clearly aligned with the previous data supporting whole-arm translocations [1,23]. However, the data also suggest the possibility of exchanges in genetic material by pericentric inversions between arms in the evolution of *Ae. aegypti* chromosomes 2 and 3.

**Figure 6:**
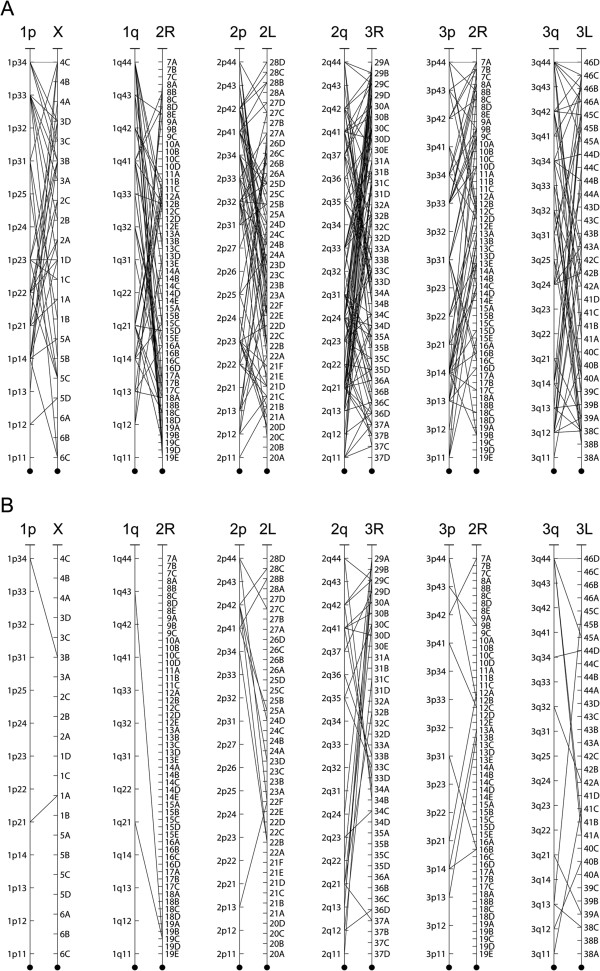
**Shuffling gene order in chromosomes of *Ae. aegypti *and *An. gambiae*.** Correspondence of **(A)** 1:1 orthologs and **(B)** microsynteny blocks are shown by lines.

In addition to the insights regarding the evolution of chromosomes between *Ae. aegypti* and *An. gambiae,* we attempted to rank the amount of gene order reshuffling within the chromosome arms (Figure 6). The gene order on the newly developed physical map of *Ae. aegypti* was compared to the gene order in the genome map of *An. gambiae*[2,16]. The genes of the *Ae. aegypti* and *An. gambiae* chromosomes were mapped based on 1:1 orthologs (Figure 6A) and microsynteny blocks (Figure 6B) at subregion resolution. A microsynteny block is defined as the sharing of nine or more orthologs between subregions of *Ae. aegypti* and *An. gambiae*. Table 2 shows the percentage of orthologs found in microsynteny blocks for each chromosome: chromosome arms 1p (15%), 1q (11%) and 3p (34%) had the lowest percentages. In agreement with previous observation of a higher rate of evolution of the 2R arm in *Anopheles*[21], these data support the idea that the sex-determining chromosome 1 and arm 3p in *Ae. aegypti* had the highest rate of gene order reshuffling*.* In particular, arm 3p in *Ae. aegypti*, homologous to arm 2R in *An. gambiae*, demonstrated the highest rate of rearrangements among the autosomes of that species. Arm 2q in *Ae. aegypti* and arm 3R in *An. gambiae* shared the greatest number of orthologs (602), microsynteny blocks (24) and percentage of orthologs within microsynteny blocks (55%), indicating that this arm had the highest degree of conservation. Although we did not attempt to normalize the data for the gene densities and number of supercontigs hybridized in each subregion, these results were still indicative of the conserved regions of the *Ae. aegypti* and *An. gambiae* genomes.

**Table 2:** **Orthologs and microsynteny blocks in the *Ae. aegypti *** **and *An. gambiae *** **genome maps**

Chromosome arms (Aa/Ag)	1p/X	1q/2R	2p/2 L	2q/3R	3p/2R	3q/3 L	total
Orthologs in synteny blocks	18	18	127	332	104	151	750
Total orthologs	120	159	332	602	308	319	1840
Percent in blocks	15%	11%	38%	55%	34%	47%	41%

Aa, *Ae. aegypti;* Ag*, An. gambiae.*

## Discussion

Availability of previously constructed physical maps for *An. gambiae*[2,15,16] and the newly developed physical map for *Ae. aegypti* allows comparison of the genomic composition of the chromosomes in two species of mosquitoes. Cytogenetic studies conducted in the past demonstrated striking differences in chromosome organization between the two mosquitoes. Despite the fact that both mosquitoes have chromosome number 2n equal to six, the length of the chromosomes and the karyotype structure are different [4]. The average length of the X chromosome and two autosomes together in malaria mosquitoes is equal to about 11 μm [39]. Malaria mosquitoes have clearly distinguishable heteromorphic sex chromosomes X and Y. These two chromosomes contain large amounts of heterochromatin. Almost half of the X chromosome is represented by heterochromatic blocks [7]. The Y chromosome is almost entirely heterochromatic. Autosomes 2 and 3 also contain large heterochromatic blocks around the centromeres. The analysis of the polytene chromosomes found three regions of intercalary heterochromatin in arms 2L, 3R and 3L in addition to pericentromeric heterochromatin [8]. Chromosomes of *Ae. aegypti* are approximately 2.3 times longer than chromosomes of *An. gambiae.* The average lengths of the chromosomes at metaphase are equal to 7.15 μm for chromosome 1, 9.46 μm for chromosome 2 and 8.36 μm for chromosome 3 [29]. Sex in *Ae. aegypti* is determined by a locus on the smallest homomorphic autosome 1 [6]. The position of heterochromatin in *Ae. aegypti* chromosomes was demonstrated using a Giemsa C-banding technique [10,40]. C-bands were originally found in pericentromeric regions of female sex-determining chromosome 1 and autosomes 2 and 3. An additional intercalary C-band was found in the female sex-determining chromosome 1. Male sex-determining chromosome 1 demonstrated no heterochromatin in two original reports. However, the presence of heterochromatin in the pericentromeric region of the male-determining chromosome has been demonstrated by a silver-staining technique [9]. Comparative studies of different strains of *Ae. aegypti*[11,40] and natural populations in Brazil [41] demonstrated polymorphism in the presence/absence and sizes of intercalary band in both male- and female-determining chromosomes 1. An additional polymorphic intercalary C-band was also found in chromosome 3 in a Brazilian population of *Ae. aegypti*[41].

**Figure 7:**
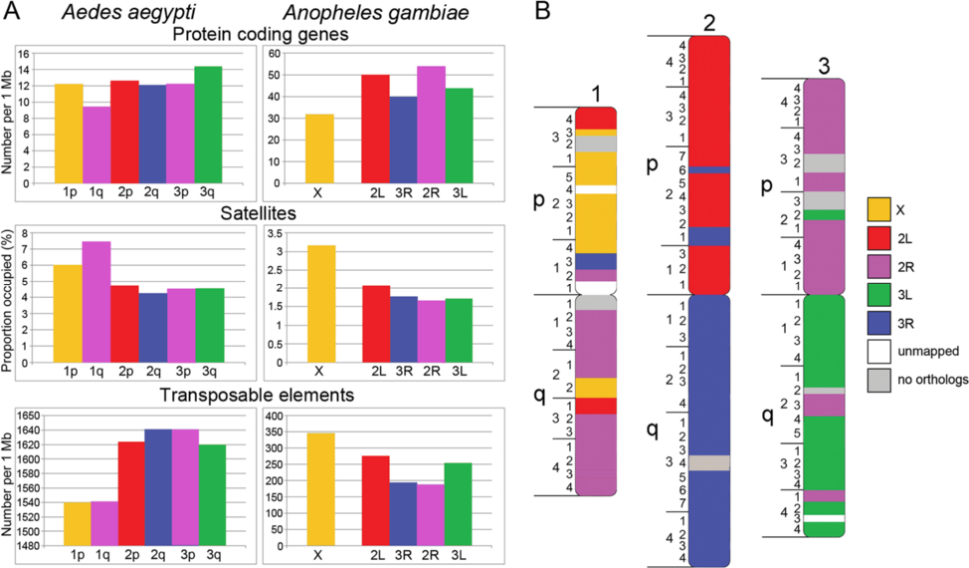
**Chromosome evolution in *Ae. aegypti *and *An. gambiae*.** Genomic composition of **(A)** protein-coding genes, satellites and transposable elements and **(B)** chromosome rearrangements between arms are shown. Chromosome arms in *An. gambiae* are represented by different colors.

The composition of genes, satellites and TEs in chromosomes of *Ae. aegypti* and *An. gambiae* is compared in Figure 7A. Gene densities were lower in sex-determining chromosome 1 in *Ae. aegypti* and in the X chromosome of *An. gambiae*. Satellites displayed an alternative pattern and were more abundant in the sex-determining chromosomes 1 of *Ae. aegypti* and X chromosome of *An. gambiae*. The distributions of genes and satellites along the chromosomes of *Ae. aegypti* (Figure 4) in general followed the C-banding patterns [10,40]. We found significantly lower density of genes and higher coverage of repeats in centromeres and in the ribosomal locus 1q22, which forms an additional C-band on the q arm of chromosome 1. The polymorphism of this band between strains and populations of *Ae. aegypti*, which has previously been demonstrated [11,40,41], can be explained by the differences in copy numbers of ribosomal genes. Interestingly, the anchor marker for the sex determination locus was mapped in the neighboring region 1q21 represented by a large condensed band on YOYO-1-stained chromosomes [14]. Nevertheless, region 1q21 had lower repeat coverage and contained a significantly higher density of genes. Similar to *D. melanogaster*[38,42], the majority of the TEs in the malaria mosquito genome were distributed in heterochromatic areas around the centromeres [8]. The TE coverage in pericentromeric heterochromatin of *An. gambiae* reached 53%. Surprisingly, TEs in *Ae. aegypti* do not follow the pattern in *An. gambiae.* By contrast, TEs in *Ae. aegypti* chromosomes are mostly spread in euchromatic regions because of their tendency to localize in gene introns [1]. According to our current estimate, the mapped portion of the *Ae. aegypti* genome contains about 5% tandem repeats and 52% TEs. Thus, the euchromatic regions of *Ae. aegypti* chromosomes have the same coverage of TEs as heterochromatin of *An. gambiae*.

The 2R arm of *An. gambiae*[1] has the highest gene densities and the lowest coverage of tandem repeats and densities of TEs [21]. Interestingly, chromosome arm 1q, which is homologous to the 2R arm of *An. gambiae*, has by contrast the lowest gene densities and the highest coverage of tandem repeats in *Ae. aegypti* (Figure 7). However, the q arm of chromosome 1 cannot be considered entirely heterochromatic as was previously suggested [28]. Even with approximately 45% of the genome placement to the chromosomes, we found that at least 594 (8%) of genes were located in this arm. The heterochromatic nature of this particular arm can be explained by the presence of the ribosomal DNA locus and the sex determination locus that can directly stimulate accumulation of the repetitive DNA. Formation of the heterochromatin around the ribosomal locus prolongs lifespan by silencing transcription of the ribosomal genes in *Drosophila*[43]. Another study conducted on *Drosophila* showed that heterochromatin controls male viability by regulating genes in the sex determination pathway [44]. Thus, our data suggest that a high coverage of satellites rather than TEs could be the major characteristic of the heterochromatin in *Ae. aegypti.* We also argue that the genomic composition of the sex-determining chromosome 1 in *Ae. aegypti* is influenced by the presence of the sex-determining locus and ribosomal genes.

Our analysis of the chromosome rearrangements revealed that the pattern of chromosome evolution between *An. gambiae* and *Ae. aegypti* was more complex than previously suggested [1]. In addition to the known exchange of genomic material between chromosome X and a part of the 2R arm of *An. gambiae* in chromosome 1 of *Ae. aegypti*, a number of pericentric inversions have reshuffled the genetic materials between chromosome arms 1p and 1q (Figure 7B). Similar patterns of pericentric inversions were also found in autosomes 2 and 3 as an addition to the previously determined whole-arm translocation [1]. Our study has also shown that gene order within chromosome arms of *Ae. aegypti* was poorly conserved, because of multiple paracentric inversion. Similar types of chromosome rearrangements have been determined in *Drosophila*[45]. For example, the metacentric X chromosomes in *D. willistoni*, *D. pseudoobscura* and *D. persimilis* were generated by a fusion of the X and autosomal 3L arm of *D. melanogaster*. Additional pericentric inversions were detected in *D. erecta*, *D. yakuba, D. pseudoobscura* and *D. persimilis* compared with the chromosome pattern of *D. melanogaster.* Similarly to the heteromorphic sex chromosomes in *Anopheles*[21] and *Drosophila*[46], homomorphic sex-determining chromosomes 1 in *Ae. aegypti* demonstrated the highest rate of the rearrangements among the chromosomes. However, unlike in *Drosophila* and *Anopheles*, chromosome 1 in *Ae. aegypti* was not enriched with TEs, which can be associated with inversion formation [47-50]. Instead, we demonstrated a high coverage of minisatellites suggesting that tandem repeats might play a special role in chromosome evolution of *Aedes* mosquitoes. Indeed, simple repeats can be involved in DNA breaks and chromosome rearrangements by the formation of hairpin and cruciform structures [51]. Thus, despite the morphological and molecular differences between homomorphic sex-determining chromosomes in *Aedes* and heteromorphic sex chromosomes in Anopheles, they both display the rapid rate of gene order evolution.

## Conclusions

This study developed a physical map comprising 45% of the yellow fever mosquito genome by assigning 624 Mb of 294 genomic supercontigs to chromosome bands. This map guided the analyses of the genes, satellites and TE landscapes in the *Ae. aegypti* genome and provided important insights into chromosome evolution in mosquitoes*.* Lower gene densities and higher satellite DNA content were detected in pericentromeric regions and region 1q22 in the homomorphic sex-determining chromosome 1, which also contains the ribosomal DNA locus. These regions can be defined as heterochromatic in *Ae. aegypti* chromosomes. In contrast to satellites, genes and TEs were more abundant in chromosomes 2 and 3, and also in euchromatic areas of the chromosomes. A comparative genomic analysis with *An. gambiae* demonstrated that the whole-arm synteny was not fully preserved. Multiple pericentric inversions substantially reshuffled the genetic material of the 1p and 1q arms. Despite this reshuffling, the homologies between 1p and X chromosome, as well as between 1q and 2R arm of *An. gambiae*, are evident. The homomorphic sex-determining chromosome 1 demonstrated the highest rate of the chromosome rearrangements. We believe that additional physical mapping is still needed to improve the current fragmented genome assembly of *Ae. aegypti*. Assignment and orientation of the supercontig assemblies of the *Ae. aegypti* genome to the chromosomes will facilitate more advanced studies of the genome organization and chromosome evolution in mosquitoes.

## Methods

### Mosquito strain

This study was performed with the Liverpool IB-12 strain of *Ae. aegypti*, which originated from the Liverpool strain following several rounds of inbreeding and was previously used for the genome sequencing project of *Ae. aegypti*[1]. The Liverpool strain has also been utilized for genetic-linkage and QTL mapping studies [17,34,35].

### Fluorescent *in situ* hybridization

Slides of mitotic chromosomes were prepared from imaginal discs of fourth instar larvae following published protocols [14,29,39]. Samples of BAC clone DNA were prepared by Clemson University Genomics Institute in 96-well plates. FISH was performed as previously described [14,39]. A two-color version of FISH was used for localizing BAC positions on mitotic chromosomes. BAC DNA for hybridization was labeled with Cy3- or Cy5-dUTP (GE Healthcare UK Ltd., Amersham, UK) by nick translation. Chromosomes were stained with YOYO-1 iodide (Invitrogen Corporation, Grand Island, NY, USA). Slides were analyzed using a Zeiss LSM 510 Laser Scanning Microscope (Carl Zeiss Microimaging, Inc., Thornwood, NY, USA) at 600× magnification.

### Genomic features analysis

For analysis of the genetic element distribution on the chromosomes, we used the sequence data from Vector Base [52] regarding each supercontig. Gene density was calculated based on the number of protein-coding genes belonging to the individual supercontigs per Mb using the BioMart tool of Vector Base [53]. Repetitive DNA content was analyzed using Tandem Repeat Finder with basic parameters [54]. Tandem repeats were defined by motif sizes from 2 to 6, from 7 to 99, and from 100 or more as microsatellites, minisatellites and satellites, respectively. Analysis of TEs was performed using Repeat Masker (version 3.2.9) [55] at the default settings using the *Ae. aegypti* TEs in TEfam database [56] as the custom repeat library. Repeat Masker output was then used to count the frequency of occurrence and the base occupied by each TE in each supercontig.

### Chromosome synteny and ortholog mapping

A collection of 5,265 previously identified orthologs in *Ae. aegypti* and *An. gambiae*[1] was downloaded from OrthoDB [57]. This collection was used to test for chromosome synteny between the two species. Among these 2,335 orthologs were located in correctly assembled supercontigs [19] within the physical map of *Ae. aegypti*. Chromosome synteny was then tested for in each subregion: each *Ae. aegypti* subregion was identified with the *An. gambiae* chromosomes sharing the greatest number of orthologs. Physical maps of 1:1 orthologs (at subregion resolution) were constructed using the physical map of *Ae. aegypti* and the genome map of *An. gambiae.* Microsynteny blocks, defined here as the sharing of nine or more orthologs between subregions of *Ae. aegypti* and *An. gambiae*, were also mapped.

## Abbreviations

BAC: bacterial artificial chromosome; bp: base pairs; FISH: fluorescent *in situ* hybridization; Mb: mega base pairs; MITEs: miniature inverted-repeat transposable elements; PCR: polymerase chain reaction; QTL: quantitative trait loci; TE: transposable element.

## Competing interests

The authors declare that they have no competing interest.

## Authors’ contributions

MVS, DWS and IVS designed experiments. VAT and BSdeB performed experiments. VAT, NAK, CM and ZT performed bioinformatics analysis. MVS, VAT, NAK and IVS wrote the manuscript. DWS provided resources. All authors read and approved the final manuscript.

## Supplementary Material

Additional file 1: Table S1 Supercontig and BAC clone positions on *Ae. aegypti* chromosomes. Major signals are indicated by asterisks. Conflict mapping data are in bold. BAC AC#, BAC clone accession number; NA, not applicable; SC, supercontig; ^ additional signals. Click here for file

Additional file 2: Figure S1 The correlation between the physical band position of a supercontig on our physical map and its cM position on the genetic linkage map [19]. Spearman’s rank correlation coefficients equal to 0.77, 0.83 and 0.65 (*P* <0.05) were determined for chromosomes **(A)** 1, **(B)** 2 and **(C)** 3, respectively. Click here for file
