# Supplementary material for: Genomic composition and evolution of Aedes aegypti chromosomes revealed by the analysis of physically mapped supercontigs
Source: BMC Biol. 2014 Apr 14;12:27. doi: 10.1186/1741-7007-12-27 (PMC4021624; doi:10.1186/1741-7007-12-27)
Supplement: Additional file 1: Table S1 — Supercontig and BAC clone positions on Ae. aegypti chromosomes. Major signals are indicated by asterisks. Conflict mapping data are in bold. BAC AC#, BAC clone accession number; NA, not applicable; SC, supercontig; ^ additional signals. [file 1741-7007-12-27-S1.docx]

**Additional file 1: Table S1 Supercontig and BAC clone positions on *Ae. aegypti* chromosomes.**

SC – supercontig, AC# - accession number, GM – genetic marker, NA – not applicable, * - major signal, ^ - additional signals, chr – chromosome, CM – centromere, potentially misassembles supercontigs are in bold.

| **SC** | **SC Size** | **BAC well/plate** | **BAC AC#1 (T7)** | **BAC AC#2 (SP6)** | **Genetic Marker** | **GM AC#** | **BAC Locations** | **SC location** |
| --- | --- | --- | --- | --- | --- | --- | --- | --- |
| 1 | 5856339 | NDL.008F24 | CC843922 | CC843917 | *Dce1* | *AF288384* | 1q33*, 2 signals on chr1 |  |
| 1 |  | NDL.027C12 | CC132566 | CC132563 | LF231 | BM005478 | 3p23 |  |
| **1** |  | NDL.067B23 | NA | NA | LF106 | BM005490 | 3p23 | **3p23** |
| 1 |  | NDL.089O7 | CC116375 | CC116371 | NA |  | 3p23 |  |
| 1 |  | NDL.089O7 | CC116375 | CC116371 | NA |  | 3p31 |  |
| 2 | 5729030 | NDL.007J20 | CC134787 | CC134784 | NA |  | 1q14 | Unknown |
| 2 |  | NDL.21C6 | CC871173 | CC871167 | NA |  | 2q21 |  |
| **3** | 5167134 | NDL.040A11 | CC113575 | CC113570 | NA |  | 3q12 | **3q12** |
| 3 |  | NDL.079O19 | CC128374 | CC128367 | NA |  | 3q42 |  |
| 3 |  | NDL.093L24 | CC125692 | CC125688 | NA |  | 3q12 |  |
| 3 |  | NDL.112J9 | CC855085 | CC855079 | NA |  | 3q11 |  |
| 3 |  | NDL.129D8 | CC863970 | CC863969 | NA |  | 3p31 |  |
| 4 | 5177111 | NDL.085K2 | NA | NA | *SiaI* | *AF108099* | 1p34 | 1p34 |
| 5 | 5058281 | NDL.044N13 | CC845577 | NA | A13L975 | BH214533 | 2p34 | 2p34 |
| 6 |  | NDL.023F4 | CC131388 | CC131382 | NA |  | 2q33 |  |
| 6 | 5075626 | NDL.131G19 | CC864061 | CC864059 | NA |  | 2q34 | 2q34 |
| **7** | 4891900 | NDL.022N5 | CC861109 | CC861104 | *MalI* | *M30442* | 3p31 | **3p31** |
| 7 |  | NDL.065J18 | CC115355 | CC115232 | NA |  | 3q44 |  |
| 7 |  | NDL.52J17 | CC859893 | CC859889 | NA |  | 3q44 |  |
| 8 |  | NDL.009K4 | CC124936 | NA | NA |  | 3q12 |  |
| 8 | 4905038 | NDL.033M5 | CC866125 | CC866020 | NA |  | 3p12 | 3p12 |
| 8 |  | NDL.127G11 | CC844150 | CC844144 | NA |  | 3p11 |  |
| 10 | 4486241 | NDL.090P20 | CC859265 | CC859263 | NA |  | 3q44 |  |
| 10 |  | NDL.107P10 | CC864644 | CC864638 | NA |  | 3q42 | 3q42 |
| 10 |  | NDL.107P10 | CC864644 | CC864638 | NA |  | 3q42 |  |
| 11 |  | NDL.029F5 | CC113808 | CC113805 | NA |  | 2q23 |  |
| 11 | 4706946 | NDL.132B17 | CC855909 | CC855903 | NA |  | 3q11 | 3q11 |
| 11 |  | NDL.53G12 | CC121288 | CC121282 | NA |  | 3q11 |  |
| 12 | 4526317 | NDL.007F16 | CC126031 | CC126029 | NA |  | 2q36 | 2q36 |
| 12 |  | NDL.032L22 | CC137309 | CC137303 | NA |  | 2q35 |  |
| 12 |  | NDL.062K20 | CC869405 | CC869401 | NA |  | 2q36 |  |
| 12 |  | NDL.131N10 | CC853904 | CC853902 | NA |  | 2q41 |  |
| 12 |  | NDL.9I16 | CC143852 | CC143845 | NA |  | 1q31 |  |
| 13 | 4376253 | NDL.089F18 | CC118989 | CC118985 | LF377 | BM005496 | 3q44 | 3q44 |
| 13 |  | NDL.090G13 | CC865050 | CC865048 | NA |  | 3q44 |  |
| 13 |  | NDL.100H16 | CC870885 | CC870882 | NA |  | 3q43 |  |
| 13 |  | NDL.120E12 | CC863986 | CC863981 | NA |  | 3q43 |  |
| 13 |  | NDL.132J4 | CC867100 | CC867094 | NA |  | 3q42 |  |
| 14 |  | NDL.022N19 | CC844954 | CC844953 | D6L600 | BH214535 | 2q42 | 2q42 |
| 14 | 4341222 | NDL.060L12 | NA | NA | ARC1 | R19561 | 2q42*, multiple signals |  |
| 15 | 4333154 | NDL.036A5 | CC868312 | CC868306 | NA |  | 2p41 | 2p41 |
| 15 |  | NDL.057N3 | CC109316 | CC109310 | NA |  | 2p41 |  |
| 16 | 4402401 | NDL.009E7 | CC131621 | NA | NA |  | 3q21 | 3q21 |
| 16 |  | NDL.023D9 | CC123559 | CC123552 | NA |  | 3q14 |  |
| 17 | 4314454 | NDL.072H24 | CC143669 | CC143666 | LF232 | BM005489 | 3p22 | 3p22 |
| 19 | 4221289 | NDL.046I8 | CC111731 | CC111728 | NA |  | 3q42*, 1p23^ | 3q42 |
| 19 |  | NDL.120B20 | CC870363 | CC870359 | NA |  | 3q42 |  |
| 20 |  | NDL.025H9 | CC117339 | CC117336 | NA |  | 1q44 |  |
| 20 | 4138018 | NDL.054P11 | CC110475 | CC110346 | NA |  | 2p44 | 2p44 |
| 20 |  | NDL.110C14 | CC853005 | CC853001 | NA |  | 2p44 |  |
| 22 | 4100794 | NDL.120J19 | CC861615 | CC861612 | NA |  | 2p34 | 2p34 |
| 23 | 3969038 | NDL.009F5 | CC143120 | CC143114 | NA |  | 3p44 | 3p44 |
| 24 | 4087421 | NDL.065G2 | CC116791 | CC116663 | NA |  | 2q24 | 2q24 |
| 24 |  | NDL.094A14 | CC142584 | CC142583 | NA |  | 2q24 |  |
| **25** | 3904351 | NDL.032A10 | CC129271 | CC129265 | NA |  | 2q37 | **2q37** |
| 25 |  | NDL.055C23 | CC845724 | CC872175 | LF138 | T58332 | 2p21 |  |
| 25 |  | NDL.073P15 | CC132641 | CC132634 | NA |  | 2q36 |  |
| 25 |  | NDL.112B9 | CC861997 | CC861993 | NA |  | 2q35 |  |
| 25 |  | NDL.123B21 | CC845086 | CC845080 | NA |  | 2q37 |  |
| 25 |  | NDL.123E21 | CC874344 | CC874341 | NA |  | 2q37 |  |
| 25 |  | NDL.125D16 | CC861711 | CC861706 | NA |  | 2q37 |  |
| 25 |  | NDL.129F10 | CC851057 | CC851051 | NA |  | 2q41 |  |
| 27 |  | NDL.027P22 | CC124931 | CC124926 | NA |  | 2q32 |  |
| 27 | 3799043 | NDL.034K11 | CC144209 | CC144203 | NA |  | 2q33 | 2q33 |
| 27 |  | NDL.040K7 | CC150939 | CC150828 | NA |  | 2q31 |  |
| 27 |  | NDL.049G15 | CC137715 | CC137708 | NA |  | 2q33*, 3p34^, 3q41^ |  |
| 27 |  | NDL.100H19 | CC849870 | CC849866 | NA |  | 2q32 |  |
| 27 |  | NDL.114F2 | CC864759 | CC864756 | NA |  | 2q35 |  |
| 27 |  | NDL.129A16 | CC868864 | CC868859 | NA |  | 2q33*, 3p33 |  |
| 28 | 3768427 | NDL.029L20 | CC127602 | CC127596 | NA |  | 2p23 | 2p23 |
| 28 |  | NDL.064H22 | CC849018 | CC849012 | NA |  | 2p24 |  |
| 28 |  | NDL.119L12 | CC846525 | CC846523 | NA |  | 2p23 |  |
| 28 |  | NDL.122H2 | CC855002 | CC854999 | NA |  | 2p26 |  |
| 29 |  | NDL.018B23 | CC122800 | CC122795 | NA |  | 2q11 |  |
| 29 |  | NDL.037A17 | CC142181 | CC142176 | NA |  | 2q42 |  |
| 29 | 3855786 | NDL.088D9 | NA | NA | *CRALBP* | *AF329893* | 2q42 | 2q42 |
| 30 | 3879053 | NDL.037P12 | CC139116 | CC139110 | NA |  | 2p34 | Unknown |
| 30 |  | NDL.104O20 | CC873709 | CC861567 | NA |  | 3p33 |  |
| 31 | 3687466 | NDL.019I15 | CC109769 | CC109763 | NA |  | 2p12 |  |
| 31 |  | NDL.023F10 | CC133317 | CC133314 | NA |  | 2q35^, 1p32^, 3p34^ |  |
| 31 |  | NDL.096K4 | CC870659 | CC845677 | NA |  | 2p13 | 2p13 |
| 31 |  | NDL.097A22 | CC872344 | CC872342 | NA |  | 2p21 |  |
| 31 |  | NDL.113P12 | CC872327 | CC872322 | NA |  | 2q34 |  |
| 31 |  | NDL.131A14 | CC844627 | CC844625 | NA |  | 2p13 |  |
| 32 | 3742797 | NDL.111D3 | CC858760 | CC858752 | NA |  | 2p41 | 2p41 |
| 33 | 3689457 | NDL.104K6 | CC849810 | CC843338 | NA |  | 1p21 |  |
| 33 |  | NDL.122D18 | CC866118 | CC866114 | NA |  | 1p22 | 1p22 |
| 34 | 3684238 | NDL.033K9 | CC844124 | CC844030 | NA |  | 2q21*, 3p32^, 3q32^ | 2q21 |
| 36 | 3595646 | NDL.067B1 | NA | CC135387 | NA |  | 2p42 | 2p42 |
| 36 |  | NDL.108H14 | CC870565 | CC873996 | NA |  | 2p42 |  |
| 38 | 3498553 | NDL.034J21 | CC136926 | CC136922 | NA |  | 2q24 | 2q24 |
| 39 | 3590018 | NDL.026O21 | CC842939 | CC842935 | LF342 | BM005512 | 2p33 | 2p33 |
| 39 | 3590018 | NDL.028E19 | CC134321 | CC134315 | NA |  | 2p33 |  |
| 39 |  | NDL.062A13 | CC852645 | CC852640 | NA |  | 3q22 |  |
| 40 | 3402887 | NDL.031L6 | CC132513 | CC132508 | NA |  | 2q41 | 2q41 |
| 40 |  | NDL.064C8 | CC847851 | CC847846 | NA |  | 2q43 |  |
| 40 |  | NDL.080C6 | CC120889 | CC120887 | NA |  | 2q41 |  |
| 41 | 3348252 | NDL.087H3 | CC112569 | CC112565 | NA |  | 2q43 | 2q43 |
| 42 | 3389925 | NDL.093E14 | CC140680 | CC140676 | NA |  | 2p22 | 2p22 |
| 42 |  | NDL.099O13 | CC842466 | CC842461 | NA |  | 2p21 |  |
| 43 | 3274742 | NDL.077N21 | CC109642 | CC109638 | NA |  | 2q36 | 2q35 |
| 43 |  | NDL.115P20 | CC844214 | CC850807 | NA |  | 2q34 |  |
| 44 |  | NDL.096I16 | CC857318 | NA | LF180 | BM005486 | 2p13 |  |
| **44** | 3232429 | NDL.100P12 | CC862851 | CC862847 | NA |  | 2q23 | **2q22** |
| 44 |  | NDL.129J12 | CC871539 | CC871536 | NA |  | 2q21 |  |
| 45 | 3214345 | NDL.018I7 | CC120539 | CC150986 | *GS1* | *AF004351* | 2q35 | 2q35 |
| 45 |  | NDL.023B17 | CC141126 | CC141119 | NA |  | 2q35 |  |
| 46 | 3321798 | NDL.008C10 | CC862644 | CC862637 | NA |  | 2p21 | 2p21 |
| 47 | 3187850 | NDL.031A5 | CC118423 | CC118420 | NA |  | 2q37 | 2q37 |
| 47 |  | NDL.072H9 | CC114300 | CC114295 | NA |  | 2q36 |  |
| 47 |  | NDL.122O17 | CC866441 | CC866435 | NA |  | 2q37 |  |
| 47 |  | NDL.123P14 | CC851113 | CC851108 | NA |  | 2q37 |  |
| 48 |  | NDL.018D3 | CC124097 | CC151019 | NA |  | 1p32 |  |
| 48 |  | NDL.074F2 | CC865086 | CC865081 | NA |  | 2q34 |  |
| 48 | 3355344 | NDL.111G22 | CC861679 | CC861676 | LF357 | BM005495 | 2q33 | 2q33 |
| 49 | 3164279 | NDL.046G23 | CC107065 | CC107060 | NA |  | 3p13 | 3p13 |
| 49 |  | NDL.100B18 | CC865034 | CC865030 | NA |  | 3p13 |  |
| 49 |  | NDL.100C17 | CC865373 | CC865368 | NA |  | 3p14 |  |
| **50** | 3200609 | NDL.009N2 | CC121343 | CC121341 | LF217 | BM005473 | 1p33 | **1p33** |
| 50 |  | NDL.034M14 | CC117063 | CC117059 | NA |  | No signal |  |
| 50 |  | NDL.093N3 | NA | CC139721 | NA |  | 3q42 |  |
| 51 | 3160936 | NDL.089L3 | CC119660 | CC119655 | NA |  | 2q41 | 2q41 |
| 51 |  | NDL.127F24 | CC860273 | CC860271 | NA |  | 2q37 |  |
| 52 | 3212514 | NDL.031K22 | CC140396 | CC140392 | NA |  | 2p42 | 2p42 |
| 54 | 3433061 | NDL.014E23 | CC122483 | CC122479 | NA |  | 1p33 | Unknown |
| 54 |  | NDL.023I17 | CC121284 | CC121279 | NA |  | 2p22 |  |
| 55 | 3141549 | NDL.032A4 | CC127388 | CC127382 | NA |  | 2q23 |  |
| 55 |  | NDL.053G23 | CC137832 | CC137828 | NA |  | 2q24 | 2q24 |
| 56 | 3188272 | NDL.131C7 | CC874209 | CC874208 | NA |  | 1q14 | 1q14 |
| 58 | 3090966 | NDL.008H13 | CC872270 | CC872267 | *MUCI* (LF398) | AF308862 | 2p32 | 2p32 |
| 58 | 3090966 | NDL.027D17 | CC136578 | CC136570 | NA |  | 2p32*, 1q22^ |  |
| 59 |  | NDL.013B13 | CC122416 | CC122412 | NA |  | 1p23 |  |
| 59 | 3045158 | NDL.033C3 | NA | NA | LF178 | T58309 | 1p31*, 2p44^ | 1p31 |
| 61 | 3227361 | NDL.060N15 | CC123692 | CC123689 | NA |  | 3q14 | 3q14 |
| 61 |  | NDL.066K5 | CC115186 | NA | NA |  | 3q13 |  |
| 62 | 3065330 | NDL.111D1 | CC858187 | CC858181 | NA |  | 2q12 | 2q12 |
| 64 | 2929083 | NDL.021J9 | CC867467 | CC867466 | NA |  | 3q33 |  |
| 64 |  | NDL.092A8 | CC129734 | CC129732 | NA |  | 3q34 | 3q34 |
| 66 | 3011136 | NDL.023H10 | CC133086 | CC133081 | NA |  | 1p14 | 1p14 |
| 66 |  | NDL.027L11 | CC112157 | CC112152 | NA |  | 1p13 |  |
| 67 | 3004230 | NDL.100I12 | CC845645 | CC845640 | NA |  | 1q32 | 1q32 |
| 68 | 2950385 | NDL.017O21 | CC845107 | CC845103 | AEGbS11 | AY033622 | 1q44 | 1q44 |
| 69 | 2946165 | NDL.009H21 | CC110496 | CC110491 | NA |  | 3q14 |  |
| 69 |  | NDL.122G17 | CC873307 | CC873303 | LF168 | R47184 | 3q12 | 3q12 |
| 70 | 2929944 | NDL.047P3 | CC114409 | CC114401 | NA |  | 1q41 | 1q41 |
| 71 | 2873990 | NDL.058C3 | CC120670 | CC120561 | *AeW* | *U73826* | 1p14 | 1p14 |
| 72 | 2994197 | NDL.027F12 | CC106921 | CC106916 | NA |  | 1p23 | 1p22 |
| 72 |  | NDL.039C9 | CC844312 | CC844306 | NA |  | 1p21 |  |
| 73 | 2899062 | NDL.113D17 | CC853989 | CC852147 | NA |  | 1q41 | 1q41 |
| 74 | 2917482 | NDL.087E16 | CC121409 | CC121404 | NA |  | 1q31 | Unknown |
| 74 |  | NDL.128P16 | CC859260 | CC859256 | NA |  | 3p41 |  |
| 75 | 2975254 | NDL.002B16 | CC866974 | CC866967 | NA |  | 1p14 | 1p14 |
| 75 |  | NDL.033E19 | CC859067 | CC858977 | NA |  | 1p14 |  |
| **76** | 2906033 | NDL.034M18 | CC133089 | CC133084 | NA |  | 2p13 | **2p13** |
| 76 |  | NDL.034M4 | CC113819 | CC113815 | NA |  | 2p12 |  |
| 76 |  | NDL.052J18 | CC848570 | CC848565 | *Chym* (LF173) | AY038039 | 2q33 |  |
| 78 | 2909025 | NDL.055L5 | CC864660 | CC862836 | NA |  | 2p32 | 2p32 |
| 80 | 2848369 | NDL.013M1 | CC143747 | CC143745 | NA |  | 1q33 | Unknown |
| 80 |  | NDL.051H16 | CC110457 | CC110451 | NA |  | 2q12 |  |
| 81 | 2864490 | NDL.127B4 | CC868179 | CC868175 | NA |  | 1q21 | 1q21 |
| 82 | 2859376 | NDL.009H22 | CC135282 | CC135277 | NA |  | 1q43 | 1q43 |
| 83 | 2974912 | NDL.097J11 | CC871492 | CC871489 | NA |  | 3q33 | 3q33 |
| 84 | 2919364 | NDL.019C8 | CC124778 | CC124773 | NA |  | 2q37 |  |
| 84 |  | NDL.110J5 | CC845941 | CC845936 | NA |  | 2q42 | 2q41 |
| 85 | 2749456 | NDL.037G23 | CC113839 | CC113835 | NA |  | 3p31 | 3p31 |
| 86 | 2716630 | NDL.019M6 | CC138832 | CC138828 | LF323 | BM005507 | 3q34 | 3q34 |
| 88 | 2707514 | NDL.046L1 | CC119582 | CC119576 | TY7 | R19560 | 1q32 | 1q32 |
| 89 |  | NDL.018N14 | CC129241 | CC129236 | NA |  | 3q25 |  |
| 89 | 2856899 | NDL.072G23 | CC106679 | CC106677 | NA |  | 3q31 | 3q31 |
| 90 | 2860138 | NDL.045H4 | CC136074 | CC136069 | NA |  | 2p12 | 2p12 |
| 90 |  | NDL.078N12 | CC138781 | CC138775 | NA |  | 3q12*, multiple signals |  |
| 91 | 2679511 | NDL.083G11 | CC131117 | CC131113 | NA |  | 3p32 | Unknown |
| 91 |  | NDL.117I16 | CC870336 | CC873985 | NA |  | 3p13 |  |
| 92 | 2802290 | NDL.035P22 | CC118171 | CC118167 | NA |  | 3q14 | 3q14 |
| 93 | 2851803 | NDL.008L17 | CC846726 | CC846722 | NA |  | 3q25 | 3q25 |
| 94 | 2735815 | NDL.033C13 | CC842609 | CC842487 | NA |  | 3p33 | 3p33 |
| 95 | 2788767 | NDL.093C11 | CC115469 | CC115464 | NA |  | 2q12 | 2q12 |
| 96 | 2767225 | NDL.010B1 | NA | NA | *slo* | *AF443282* | 1q33 | 1q33 |
| 96 |  | NDL.087M5 | CC108368 | CC108365 | NA |  | 1q33 |  |
| 98 |  | NDL.018C3 | CC111998 | CC150479 | NA |  | 2q23 |  |
| **98** | 2816416 | NDL.051L10 | CC116032 | CC116025 | *def* | *AF156088* | 3q32*, multiple signals | **3q32** |
| 99 | 2679511 | NDL.049M20 | CC133516 | CC133511 | NA |  | 3p32 |  |
| 99 |  | NDL.097N12 | CC866880 | CC866872 | NA |  | 1p25 | Unknown |
| 101 | 2937510 | NDL.018I10 | CC108444 | CC151380 | NA |  | 2q33 |  |
| 101 |  | NDL.110I23 | CC853425 | CC853419 | NA |  | 2q31 |  |
| 101 |  | NDL.128O17 | CC860389 | CC860382 | NA |  | 2q32 | 2q32 |
| 103 | 2623108 | NDL.051M2 | CC123870 | NA | NA |  | 2q22 | 2q21 |
| 103 |  | NDL.073J5 | CC122200 | CC122191 | NA |  | 2q13 |  |
| 105 | 2568089 | NDL.024I4 | CC126391 | CC126387 | NA |  | 3p14 | 3p14 |
| 105 |  | NDL.070K2 | CC122925 | CC122917 | NA |  | 3p13 |  |
| 107 | 2543601 | NDL.033C11 | CC857055 | CC856942 | NA |  | 3p42 | 3p42 |
| 108 | 2638595 | NDL.120L15 | CC847137 | CC847133 | NA |  | 2q22 | 2q22 |
| 109 | 2772702 | NDL.041B24 | CC862340 | CC853768 | NA |  | 2q24 | 2q24 |
| 111 | 2732153 | NDL.114O22 | CC852519 | CC852513 | NA |  | 1p21 | 1p21 |
| 112 | 2520184 | NDL.004P14 | CC142595 | CC142590 | NA |  | 2p31 | 2p31 |
| 112 |  | NDL.022C6 | CC859287 | CC859281 | NA |  | 2p27 |  |
| 113 | 2495981 | NDL.058C7 | CC125558 | CC125439 | NA |  | 2q21 |  |
| 113 |  | NDL.097M1 | CC864574 | CC864570 | NA |  | 2q13 | 2q13 |
| 114 |  | NDL.008O21 | CC856895 | CC856893 | NA |  | 2q35 |  |
| 114 | 2490165 | NDL.027N12 | CC121937 | CC121932 | NA |  | 2q33 | 2q34 |
| 115 | 2479458 | NDL.034L3 | CC116137 | CC116135 | NA |  | 3q33 | 3q34 |
| 115 |  | NDL.049I18 | CC110634 | CC110630 | NA |  | 3q42 |  |
| 117 | 2495114 | NDL.039D4 | CC841980 | CC841978 | NA |  | 1q33 | 1q33 |
| 118 |  | NDL.014D24 | NA | CC136873 | NA |  | 1p33* | 1p33 |
| 118 | 2484251 | NDL.045L10 | CC122791 | CC122787 | NA |  | 1p32 |  |
| 119 | 2438940 | NDL.034A20 | CC134001 | CC133996 | NA |  | 3q11 | 3q11 |
| 119 |  | NDL.095F13 | CC138815 | CC138810 | NA |  | 3q11 |  |
| 120 | 2427180 | NDL.099K22 | CC860108 | CC860103 | NA |  | 2p32 | 2p32 |
| 121 | 2378472 | NDL.005F4 | CC134023 | CC108913 | NA |  | 2q42 | 2q42 |
| 121 |  | NDL.080A13 | CC113658 | CC113656 | NA |  | 2q43 |  |
| 122 |  | NDL.014B6 | CC131295 | CC131290 | NA |  | 2p23 |  |
| 122 | 2401221 | NDL.052E23 | CC844284 | CC844276 | *Sec61* | *AF326338* | 2q24 | 2q24 |
| 123 | 2410060 | NDL.017H6 | CC867073 | CC867068 | LF179 | BM005479 | 1p12 | 1p12 |
| 123 |  | NDL.100G8 | CC857841 | CC857837 | LF314 | BM005509 | 1p12 |  |
| 124 | 2397010 | NDL.066C6 | CC110219 | CC110215 | NA |  | 2p41 | 2p41 |
| 127 | 2370492 | NDL.123J4 | CC841926 | CC841918 | NA |  | 1p21 | 1p21 |
| 128 | 2297307 | NDL.029F24 | CC121699 | CC121696 | NA |  | 2q33 | 2q33 |
| 129 | 2318608 | NDL.056G21 | CC873317 | CC873311 | NA |  | 2q21 | 2q21 |
| 131 | 2264367 | NDL.009L15 | CC116071 | CC116065 | NA |  | 3q21 | 3q21 |
| 131 |  | NDL.051I12 | CC119573 | CC119567 | NA |  | 3q22 |  |
| 132 | 2355619 | NDL.008I12 | CC869678 | CC869676 | LF233 | T58327 | 2p32 | 2p32 |
| 133 | 2339188 | NDL.001C19 | CC134554 | CC134552 | NA |  | 1q43 | 1q43 |
| 134 | 2272101 | NDL.120J21 | CC862197 | CC862192 | NA |  | 2p32 | 2p32 |
| 135 | 2306847 | NDL.042C15 | CC136995 | CC136989 | NA |  | 2q24 | 2q24 |
| 136 | 2223392 | NDL.023A10 | CC113750 | CC113742 | NA |  | 3q12 | 3q12 |
| 136 |  | NDL.072F16 | CC118529 | CC118527 | NA |  | 3q12 |  |
| 137 | 2239969 | NDL.083A9 | CC123263 | CC123261 | NA |  | 2p21 | 2p21 |
| 139 | 2181600 | NDL.007K11 | CC117851 | CC117847 | NA |  | 2q41 | 2q41 |
| 141 | 2185411 | NDL.011F1 | NA | NA | LF111 | BM005492 | 3p33*, multiple signals | 3p33 |
| 143 | 2167940 | NDL.034J9 | CC133051 | CC133046 | NA |  | 3q32 | 3q32 |
| 145 | 2217386 | NDL.106A1 | CC854931 | NA | AEGI8 | AF326340 | 2p44 | 2p44 |
| 146 |  | NDL.013B2 | CC143387 | CC143383 | NA |  | 3p32 |  |
| 146 | 2147469 | NDL.030K18 | CC863884 | CC863880 | LF253 (LF315) | T58331 | 3p31 | 3p31 |
| 147 | 2138830 | NDL.108G7 | CC865375 | CC872784 | NA |  | 1q13 | 1q13 |
| 148 |  | NDL.030J23 | CC847424 | CC847420 | LF98 | T58313 | 2p41 |  |
| 148 |  | NDL.054P6 | CC114870 | CC114763 | NA |  | 1p34 |  |
| **148** | 2163576 | NDL.088A20 | NA | NA | LF90 | T58320 | 1p34 | **1p34** |
| 149 | 2106025 | NDL.027J11 | CC127129 | CC127125 | NA |  | 2q21 | 2q21 |
| 149 |  | NDL.045E8 | CC140308 | CC140302 | NA |  | 2q21 |  |
| 150 | 2221698 | NDL.087E8 | CC141589 | CC141585 | LF417 | BM005499 | 3p33 | 3p33 |
| 151 | 2103471 | NDL.118J5 | NA | CC847928 | AEGI27 (LF181) | BG937399 | 2p41 | 2p41 |
| 152 | 2109071 | NDL.028D13 | CC107188 | CC107181 | NA |  | 2q31*, 2q13^ | 2q31 |
| 153 | 1723990 | NDL.059H24 | NA | CC119588 | D6L500 | BH214541 | 1q33 | 1q33 |
| 154 | 2089133 | NDL.106K14 | CC860163 | CC849855 | NA |  | 2q12 | Unknown |
| 154 |  | NDL.132P2 | CC859792 | CC859791 | NA |  | 3q13 |  |
| 155 |  | NDL.001A1 | CC128923 | CC128920 | NA |  | 1q32 |  |
| 155 | 2087417 | NDL.042B22 | CC115829 | CC115825 | NA |  | 1q32 | 1q32 |
| 156 |  | NDL.006D22 | CC118810 | NA | NA |  | Multiple signals |  |
| 156 | 2106494 | NDL.070I5 | CC114410 | CC114402 | NA |  | 1p22 | 1p22 |
| 157 |  | NDL.043O14 | CC110023 | CC150194 | NA |  | 2q23-24 |  |
| 157 | 2064759 | NDL.045M21 | CC126996 | CC126992 | LF203 | BM005503 | 2q21 | 2q21 |
| 158 | 2048688 | NDL.123A9 | CC850957 | CC850955 | NA |  | 1p34 | 1p34 |
| 159 | 2117153 | NDL.043O5 | CC151681 | NA | LF396 | BM005498 | 3p34 | 3p34 |
| 161 | 2063009 | NDL.013H1 | CC111013 | CC111010 | NA |  | 1p33 | 1p33 |
| 161 |  | NDL.060H20 | CC142507 | CC142505 | NA |  | 1p34 |  |
| 163 | 2030282 | NDL.049L22 | CC123808 | CC123802 | NA |  | 3q44 | 3q44 |
| 164 | 2039552 | NDL.001A8 | CC118219 | CC118217 | NA |  | 3p14 | 3p14 |
| 164 |  | NDL.059N15 | CC136998 | CC136993 | NA |  | 3p14 |  |
| 165 |  | NDL.044G4 | CC847555 | CC847550 | NA |  | 3p43 | 3p42 |
| 165 | 1992430 | NDL.116E24 | CC870947 | CC849647 | NA |  | 3p41 |  |
| 166 | 2012222 | NDL.075D22 | CC108808 | CC108803 | NA |  | 1p25 | 1p25 |
| 167 | 2081902 | NDL.053F21 | CC126787 | CC126784 | NA |  | 2p22 | 2p22 |
| 168 | 1992380 | NDL.005G11 | CC137000 | CC131441 | NA |  | 2q35 | 2q35 |
| 168 |  | NDL.023E20 | CC139240 | CC139236 | NA |  | 2q34* |  |
| 169 | 1997338 | NDL.079L4 | CC137908 | CC123778 | NA |  | 2p25 | 2p25 |
| 170 | 2080708 | NDL.042F21 | CC135990 | CC135986 | NA |  | 2q24 | 2q24 |
| 170 |  | NDL.121C2 | CC849408 | CC849403 | NA |  | 2q31 |  |
| 171 | 2025736 | NDL.056I6 | CC844161 | CC844160 | NA |  | 3q11 | 3q11 |
| 172 | 2090550 | NDL.065O9 | CC139923 | CC137251 | NA |  | 2q22 | 2q22 |
| 173 | 1959865 | NDL.044L19 | CC873776 | CC873773 | NA |  | 2q42 | 2q42 |
| 173 |  | NDL.129P12 | CC844699 | CC844693 | NA |  | 3q37*, CM chromosome 3 |  |
| 174 | 1986401 | NDL.119C7 | CC873501 | CC852759 | NA |  | 2p22 | 2p22 |
| 176 | 2016409 | NDL.023E9 | CC133926 | CC133920 | NA |  | 1p34 | 1p34 |
| 177 | 1976150 | NDL.043A19 | CC139997 | CC139992 | NA |  | 3q12 | 3q12 |
| 179 | 1915443 | NDL.014J19 | CC140766 | CC140761 | LF296 | BM005501 | 3p14 | 3p14 |
| 181 | 1928061 | NDL.124I18 | CC856659 | CC856657 | NA |  | 1p33 | 1p33 |
| 182 |  | NDL.028K9 | CC123604 | CC123598 | NA |  | 3q14 |  |
| 182 | 1961869 | NDL.118P5 | CC874663 | CC874659 | NA |  | 3q14 | 3q14 |
| 183 |  | NDL.014I5 | CC124205 | CC124200 | NA |  | 2p44 |  |
| 183 | 1899110 | NDL.097I23 | CC844063 | CC844059 | NA |  | 2p44 | 2p44 |
| 184 | 2028366 | NDL.032C18 | CC131654 | CC131651 | NA |  | 3p13 |  |
| 184 |  | NDL.037J1 | CC126570 | CC126564 | NA |  | 3p14 | 3p14 |
| 185 | 1913756 | NDL.118F7 | CC862086 | CC862082 | NA |  | 2q44 | 2q44 |
| 186 | 1957664 | NDL.007B21 | CC133183 | CC133177 | NA |  | 3q31 |  |
| 186 | 1957664 | NDL.056P6 | CC848131 | CC848127 | *para* | *AF468968* | 3q25 | 3q25 |
| 187 | 1975842 | NDL.050P12 | CC114744 | CC114739 | NA |  | 3p33 | 3p33 |
| 189 |  | NDL.007M11 | CC140361 | CC140356 | NA |  | 3q24 |  |
| 189 | 2189733 | NDL.025N11 | CC110426 | CC110421 | NA |  | 3q24 | 3q24 |
| 192 | 1864021 | NDL.018P1 | CC123174 | NA | *LAP* | *M95187* | 1p13 | 1p13 |
| 194 | 1869119 | NDL.081B14 | CC137246 | CC137245 | NA |  | 3p14 | 3p14 |
| 196 | 1880505 | NDL.051F20 | CC126760 | CC126753 | NA |  | 2q11 | 2q11 |
| 197 | 1881039 | NDL.017O2 | CC848239 | CC848236 | LF96 | BM005491 | 3p34 | 3p34 |
| 199 | 1892278 | NDL.053F17 | CC125505 | CC125499 | NA |  | 1p13 | 1p13 |
| 200 | 1852562 | NDL.023K16 | CC130380 | CC130375 | NA |  | 2p25 | 2p25 |
| 201 |  | NDL.001D5 | CC127324 | CC127319 | NA |  | 3q44 |  |
| 201 | 1818773 | NDL.116G24 | CC869734. | CC869731 | *Apy1* | *L12389* | 3q44 | 3q44 |
| 202 | 1832682 | NDL.118A24 | CC850966 | CC850962 | NA |  | 2q32 | 2q32 |
| 203 | 1808864 | NDL.019M24 | CC121915 | CC121912 | NA |  | 3q13 | 3q13 |
| 204 |  | NDL.047P21 | CC134962 | CC134957 | NA |  | 2q37 |  |
| 204 | 1827752 | NDL.048J19 | CC113737 | NA | *D7* | *M33156* | 2q36 | 2q36 |
| 205 | 1812159 | NDL.089O3 | CC115068 | CC115065 | NA |  | 2q41 | 2q41 |
| 206 |  | NDL.032L19 | CC111885 | CC111878 | NA |  | 1p25 |  |
| **206** | 1835550 | NDL.057G20 | NA | NA | a12 | BH214530 | 3q13 | **3q13** |
| 208 |  | NDL.049J18 | CC122753 | CC122748 | NA |  | 3p44 |  |
| 208 | 1789578 | NDL.050N15 | CC143821 | CC151151 | NA |  | 3p42 | 3p42 |
| 209 |  | NDL.005A24 | CC135680 | NA | LF92 | BM005493 | 3p32*, multiple signals |  |
| **209** | 1779872 | NDL.045E5 | CC114840 | CC114837 | LF211 | BM005514 | 2q44 | **2q44** |
| 210 | 2048554 | NDL.061G1 | CC115347 | CC115217 | *VCP* | *L46594* | 2p42 | 2p42 |
| 211 | 1879041 | NDL.093B5 | CC140646 | CC140644 | NA |  | 2p25 | 2p25 |
| 211 |  | NDL.094D22 | CC119500 | CC119499 | NA |  | 2p25 |  |
| 213 | 1750228 | NDL.025H18 | CC107171 | CC107168 | F17M590 | BH214537 | 2q44 | 2q44 |
| 213 |  | NDL.057G4 | CC114780 | CC114777 | NA |  | 2q42 |  |
| 216 |  | NDL.025P18 | CC136944 | CC136942 | NA |  | 2q32 |  |
| 216 | 1816664 | NDL.041C6 | CC855795 | CC855697 | *VMP15a-3* | *U91682* | 2q31 | 2q31 |
| 217 | 1823077 | NDL.008B14 | CC854507 | CC854500 | *RpL31* | *AF324863* | 3q34 | 3q34 |
| 218 | 1785985 | NDL.005C6 | CC137623 | CC113916 | NA |  | 1q12 | 1q12 |
| 218 |  | NDL.008J2 | CC864441 | CC864435 | NA |  | 1q12 |  |
| 219 |  | NDL.015J1 | NA | NA | LF407 | BM005510 | 2p34 |  |
| 219 | 1777957 | NDL.020N18 | CC118768 | CC118763 | *AmyI* | *AF000569* | 2p34 | 2p34 |
| 221 | 1752526 | NDL.089M4 | CC117391 | CC117386 | *CYP9J2* | *AF329892* | 3q41 | 3q41 |
| 223 | 1749832 | NDL.061M18 | CC142846 | CC142714 | NA |  | 3p44 |  |
| 223 |  | NDL.072N6 | CC130240 | CC130237 | NA |  | 3p41 | 3p41 |
| 226 | 1779605 | NDL.079I15 | CC119901 | CC119897 | NA |  | 2q24 | 2q24 |
| 227 | 1699844 | NDL.078K24 | CC122855 | CC122851 | NA |  | 3p43 | 3p43 |
| 229 | 1798060 | NDL.056E8 | CC858185 | CC858180 | NA |  | 3p42 | 3p42 |
| 230 |  | NDL.014N18 | CC112205 | CC112201 | NA |  | 2q35 |  |
| 230 | 1726262 | NDL.054E20 | CC128247 | CC128128 | NA |  | 2q36 | 2q36 |
| 231 | 1750236 | NDL.023F5 | CC107231 | CC107225 | NA |  | 3p13 | 3p13 |
| 232 | 1763874 | NDL.002C16 | CC855965 | CC855964 | NA |  | 1p12 | 1p12 |
| 233 | 1709798 | NDL.081L22 | CC131801 | CC131794 | NA |  | 3q24 | 3q24 |
| 236 |  | NDL.068L1 | CC126098 | CC125989 | NA |  | 2p23 |  |
| 236 | 1704329 | NDL.075E3 | CC111389 | CC111387 | NA |  | 2p23 | 2p23 |
| 237 | 1754865 | NDL.079D16 | CC126789 | CC132151 | NA |  | 1q11*,2q12^,3p21^, 3q23^ |  |
| 237 |  | NDL.092K4 | CC143271 | CC143266 | NA |  | 3q23 | 3q23 |
| 238 | 1698524 | NDL.007E2 | CC132145 | CC132140 | NA |  | 2q13 | 2q13 |
| 238 |  | NDL.050N22 | CC132941 | CC132934 | NA |  | Multiple signals |  |
| 239 |  | NDL.012H23 | CC108655 | CC108651 | NA |  | 2q41 | 2q41 |
| 239 | 1627255 | NDL.065G4 | CC137975 | CC137843 | NA |  | 2q42 |  |
| 240 | 1807018 | NDL.123N18 | CC865741 | CC865736 | NA |  | 1p12 | 1p12 |
| 241 | 1703019 | NDL.025P12 | CC135007 | CC135001 | NA |  | 2p24 | 2p24 |
| 241 |  | NDL.058C21 | NA | CC124157 | NA |  | 2p24 |  |
| 242 | 1687076 | NDL.068N18 | CC131444 | CC131322 | NA |  | 2q22 | 2q22 |
| 243 | 1637354 | NDL.019J3 | CC118032 | CC118026 | NA |  | 2p44 | Unknown |
| 243 |  | NDL.024A4 | CC134137 | CC134132 | NA |  | 2q33 |  |
| 244 | 1610334 | NDL.044A23 | CC850916 | NA | LF335 | BM005505 | 2q41 | 2q41 |
| 246 | 1607634 | NDL.106I2 | CC856950 | CC843631 | NA |  | 2q23 | 2q23 |
| 247 | 1597882 | NDL.016M12 | CC129844 | CC129839 | NA |  | 3p12 |  |
| 247 |  | NDL.072A2 | CC117193 | CC117188 | NA |  | 3p11 | 3p11 |
| 248 | 1685694 | NDL.060C13 | CC118928 | CC118926 | NA |  | 2q11 | 2q11 |
| 250 | 1628417 | NDL.083J16 | CC116855 | CC116852 | NA |  | 3p21 | 3p21 |
| 251 | 1562530 | NDL.128C1 | CC842977 | CC842972 | NA |  | 2q11 | 2q11 |
| 252 | 1852562 | NDL.035O21 | CC118789 | NA | *FerH* | *AF326341* | 1q41 | 1q41 |
| 253 | 1632056 | NDL.079L10 | CC139846 | CC112378 | NA |  | 3q44 | 3q44 |
| 255 | 1580955 | NDL.098K20 | CC873052 | CC873048 | NA |  | 2p25 | 2p25 |
| 256 | 1556621 | NDL.007K3 | CC115279 | CC115278 | NA |  | 2q21 | 2q21 |
| 259 | 1579804 | NDL.032G14 | CC137886 | CC137879 | NA |  | 2p11 |  |
| 259 |  | NDL.064G23 | CC850097 | CC850091 | a14 | BH214531 | 2p11 | 2p11 |
| 260 | 1562005 | NDL.116C10 | CC845924 | CC845923 | NA |  | 1q21 | 1q21 |
| 261 | 1569243 | NDL.100B21 | CC857226 | CC857220 | NA |  | Multiple signals | Unknown |
| 263 | 1557198 | NDL.124F24 | CC862493 | CC862491 | NA |  | 2q13 | 2q13 |
| 264 | 1569243 | NDL.109B20 | CC857183 | CC857082 | NA |  | No signal | Unknown |
| 265 | 1623187 | NDL.006D5 | CC111594 | NA | NA |  | Multiple signals | Unknown |
| 265 |  | NDL.016G5 | CC133399 | CC133395 | NA |  | Multiple signals |  |
| 266 | 1514738 | NDL.127N23 | CC864736 | CC864731 | NA |  | 3p44 | 3p44 |
| 267 | 1546120 | NDL.004P11 | CC117160 | CC117157 | NA |  | 1p31 | 1p31 |
| 267 |  | NDL.036F14 | CC856461 | CC856456 | NA |  | Unmapped |  |
| 268 | 1568215 | NDL.028P14 | CC109004 | CC109001 | NA |  | 2p27 | 2p27 |
| 268 |  | NDL.051L12 | CC116693 | CC116687. | NA |  | 2p33 |  |
| 269 | 1593547 | NDL.131M6 | CC842062 | CC842060 | NA |  | 2q34 | 2q34 |
| 271 | 1544749 | NDL.073I18 | CC138747 | CC138743 | NA |  | 1p34 | 1p34 |
| 273 |  | NDL.060F1 | CC112176 | CC112174 | NA |  | 2q35 | 2q35 |
| 273 | 1503418 | NDL.082I17 | CC843210 | CC843203 | NA |  | 2q35 |  |
| 274 | 1490669 | NDL.008M2 | CC873475 | CC873474 | NA |  | 2q12 | 2q12 |
| 275 | 1521713 | NDL.098E9 | CC861874 | CC861870 | LF223 | BM005515 | 2q44 | 2q44 |
| 276 | 1475209 | NDL.083O15 | CC124711 | CC124706 | NA |  | 3p14 | 3p14 |
| 277 | 1632801 | NDL.097L13 | CC858714 | CC858708 | LF282 | T58328 | 2p32 | 2p32 |
| 279 | 1534177 | NDL.079P18 | CC127159 | CC127152 | NA |  | 1q31 | 1q31 |
| 281 | 1451347 | NDL.001P15 | CC107550 | CC107546 | NA |  | 1q42 | 1q42 |
| 284 | 1500899 | NDL.053G17 | CC135893 | CC135888 | NA |  | 2q13 | 2q13 |
| 284 |  | NDL.114N22 | CC843415 | CC843410 | NA |  | 2q13 |  |
| 286 |  | NDL.004C10 | CC114700 | CC114694 | NA |  | 1q32 | Unknown |
| 286 | 1444863 | NDL.056E22 | CC842011 | CC842005 | NA |  | 2p44 |  |
| 287 | 1460099 | NDL.125A19 | CC843467 | CC843462 | NA |  | 3q42 | 3q42 |
| 288 | 1557290 | NDL.069O7 | CC120822. | CC120819 | NA |  | 1p25 | Unknown |
| 288 |  | NDL.083P15 | CC136763 | CC136757 | NA |  | 2q44 |  |
| 290 | 1591575 | NDL.045K4 | CC108720 | CC108716 | AEGI10 | BI096854 | 2q22 | 2q22 |
| 292 | 1406973 | NDL.132J22 | CC872215 | CC872211 | NA |  | 2q42 | 2q42 |
| 295 | 1404970 | NDL.081P22 | CC139322 | CC139319 | NA |  | 1q22 | 1q22 |
| 300 | 1467236 | NDL.055H21 | CC862564 | CC860773 | NA |  | 3p11 | 3p11 |
| 301 |  | NDL.001O10 | CC141081 | CC141078 | NA |  | 3p43 |  |
| 301 | 1424203 | NDL.041B18 | CC847484 | NA | LF347 | T58329 | 3p44 | 3p44 |
| 302 | 1420414 | NDL.043A10 | CC109256 | CC109251 | NA |  | 3p23 | Unknown |
| 302 |  | NDL.051I8 | CC118310 | CC118304 | NA |  | 1p12 |  |
| 303 | 1418205 | NDL.055J21 | CC849160 | CC853835 | NA |  | 3q23 | 3q23 |
| 304 | 1395067 | NDL.088N4 | NA | NA | LF128 | BM005494 | 3p43 | 3p43 |
| 305 | 1379243 | NDL.128P22 | NA | NA | LF227 | T58323 | 3p34 | 3p34 |
| 306 | 1398852 | NDL.021P14 | CC863757 | CC863755 | NA |  | 2p44 | 2p44 |
| 309 | 1345217 | NDL.041O14 | CC856731 | CC856631 | NA |  | 3q23 | 3q23 |
| 312 | 1439306 | NDL.114F11 | CC858658 | CC858654 | NA |  | 1p32 | 1p32 |
| 313 | 1369903 | NDL.065G15 | CC122186 | CC124249 | NA |  | 1p21 | 1p21 |
| 314 | 1358904 | NDL.098I18 | CC872639 | CC872635 | NA |  | 2q21 | 2q21 |
| 315 | 1341213 | NDL.101N16 | CC865500 | CC865496 | NA |  | 1q32 | 1q32 |
| 316 | 1322148 | NDL.036I21 | NA | NA | B8M980 | BH214534 | 2q37 | 2q37 |
| 318 | 1339553 | NDL.026C8 | CC848009 | CC848005 | NA |  | 1p23 | 1p23 |
| 319 | 1366586 | NDL.014P21 | CC111154 | CC111149 | *Rdl* | *U28803* | 2p43 | 2p43 |
| 320 | 1307916 | NDL.012M19 | CC140081 | CC140078 | NA |  | 3p41 | 3p41 |
| 321 | 1317566 | NDL.044J23 | CC854857 | CC854853 | NA |  | 3q13 | 3q13 |
| 323 | 1316576 | NDL.124E20 | CC850472 | CC850470 | NA |  | 1q13 | 1q13 |
| 326 | 1381451 | NDL.058D8 | CC142841 | CC142705 | *AmyII* | *AF000568* | 1q12 | 1q12 |
| 328 |  | NDL.017L9 | CC865977 | CC865974 | NA |  | 3q32 |  |
| **328** | 1305728 | NDL.044H3 | CC869613 | NA | LF115 | R67978 | 2p42 | **2p42** |
| 329 | 1327481 | NDL.016G18 | CC139366 | CC139360 | NA |  | 2q36 | 2q36 |
| 332 | 1291250 | NDL.016B21 | CC135433 | CC135429 | NA |  | 2p23 | 2p23 |
| 334 | 1263900 | NDL.045B9 | CC120733 | CC120728 | NA |  | 2q31 | 2q32 |
| 334 |  | NDL.099M12 | CC864163 | CC864156 | NA |  | 2q33 |  |
| 337 | 1377315 | NDL.132J2 | CC853307 | CC853303 | NA |  | 2q43 | 2q43 |
| 338 | 1244558 | NDL.054L1 | CC132265 | CC132162 | NA |  | 2p12 | 2p12 |
| 340 | 1254338 | NDL.013O2 | CC115972 | NA | NA |  | 2p24 | 2p24 |
| 341 | 1236773 | NDL.072G3 | CC123034 | CC123032 | NA |  | 2p44 | 2p44 |
| 341 |  | NDL.095H5 | CC144056 | CC144049 | NA |  | 2p44 |  |
| 342 | 1344886 | NDL.131B4 | CC852408 | CC852405 | NA |  | 2q23 | 2q23 |
| 346 | 1247450 | NDL.037K15 | CC118783 | CC118778 | NA |  | 1q11 | 1q11 |
| 346 |  | NDL.079B8 | CC124469 | CC124465 | NA |  | Multiple signals |  |
| 352 | 1201835 | NDL.121K12 | CC845466 | CC845461 | NA |  | 2q11 | 2q11 |
| 356 | 1286069 | NDL.050D10 | CC137090 | CC137088 | NA |  | 2p43 | 2p43 |
| 358 | 1313945 | NDL.109D6 | CC851350 | CC851243 | NA |  | 1p25 | 1p25 |
| 360 | 1233783 | NDL.031O18 | CC109056 | CC109051 | NA |  | 1q11 |  |
| 360 |  | NDL.039L22 | CC853309 | CC853306 | NA |  | 1p23 | 1p23 |
| 364 | 1162622 | NDL.041M14 | CC863481 | CC863374 | NA |  | 2p23 | 2p23 |
| 365 | 1161285 | NDL.115D12 | CC843304 | CC869102 | NA |  | 3p41 | 3p41 |
| 366 | 1160760 | NDL.132O12 | CC865090 | CC865085 | NA |  | 2q13 | 2q13 |
| 368 | 1193203 | NDL.100L3 | CC851960 | CC851957 | NA |  | 2p44 | 2p44 |
| 370 | 1154612 | NDL.018E13 | CC114953 | CC150510 | NA |  | 1q31 | 1q31 |
| 371 | 1152622 | NDL.023B7 | CC137913 | CC137909 | NA |  | 2q37 | 2q37 |
| 371 |  | NDL.072B17 | CC109622 | CC109618 | NA |  | 2q37 |  |
| 372 | 1227132 | NDL.098F24 | CC845170 | CC845166 | NA |  | 2q31 | 2q31 |
| 373 | 1210988 | NDL.098P15 | CC867266 | CC867264 | NA |  | 1p13 | 1p13 |
| 376 | 1159017 | NDL.002E21 | CC855646 | CC855641 | NA |  | 3p12 | 3p12 |
| 377 | 1204331 | NDL.061B12 | CC111992 | CC111871 | AEGI23 | AY033624 | 2p32 | 2p32 |
| 379 | 1140138 | NDL.026H3 | CC842274 | CC842269 | NA |  | 1q31 | 1q31 |
| 380 | 1220987 | NDL.049A15 | CC130420 | CC130418 | NA |  | 2p11 | 2p11 |
| 384 | 1174792 | NDL.026H12 | CC846383 | CC846381 | NA |  | 1p32 | 1p32 |
| 385 | 1140532 | NDL.035C21 | CC119034 | CC119029 | NA |  | 2p26 | 2p26 |
| 386 | 1156810 | NDL.037K17 | CC119411 | CC119406 | LF316 | BM005516 | 3p42*, multiple signals | 3p42 |
| 388 | 1135229 | NDL.073M10 | CC143725 | CC143723 | LF159 | T58315 | 1q21 | 1q21 |
| 392 | 1158357 | NDL.046H5 | CC136101 | CC136096 | NA |  | 3q25 | 3q25 |
| 395 | 1152404 | NDL.107N17 | CC871358 | CC871355 | NA |  | 2q11 | 2q11 |
| 410 | 1186358 | NDL.055A19 | CC857922 | CC874799 | NA |  | 2p31 | 2p31 |
| 415 | 1112054 | NDL.044O22 | CC858922 | NA | LF204 | BM378050 | 1p21 | 1p21 |
| 426 | 1138293 | NDL.072D22 | CC135451 | CC135444 | NA |  | 2q36 | 2q36 |
| 438 | 1121788 | NDL.054F19 | CC121540 | CC121419 | AEGI22 | BI099650 | 1q44 | 1q44 |
| 440 | 967717 | NDL.060C14 | CC150449 | CC143780 | RT6 | BH214544 | 1q44 | 1q44 |
| 441 | 1009977 | NDL.073A24 | CC125690 | CC125687 | *apoLp-II* | *AF038654* | 3p32 | 3p32 |
| 446 | 1002973 | NDL.062O7 | NA | NA | LF284 | BM005502 | 1q21 | 1q21 |
| 456 | 945809 | NDL.039N24 | CC873974 | CC873972 | *SDR* | *AY0033621* | 2q13 | 2q13 |
| 470 | 908159 | NDL.008N7 | CC863890 | CC863888 | *PABP* | *AY038043* | 3q44 | 3q44 |
| 477 | 943092 | NDL.067K5 | NA | NA | *AEG12* | *AY038041* | 2q31 | 2q31 |
| 506 | 866177 | NDL.132I16 | CC859722 | CC859720 | LF108 | T58322 | 3q22 | 3q22 |
| 507 | 845243 | NDL.036A1 | CC867152 | CC867148 | LF275 | BM005500 | 2q23 | 2q23 |
| 581 | 760982 | NDL.041P5 | CC875159 | CC875155 | LF250 | T58310 | 2p42 | 2p42 |
| 650 | 647669 | NDL.050A4 | CC138100 | CC138096 | *NaK* | *AF393727* | 1q42 | 1q42 |
| 673 | 598027 | NDL.129I16 | CC861928 | CC861925 | *CHT2* | *AF026492* | 1q13 | 1q13 |
| 701 | 988662 | NDL.091P10 | CC140597 | CC140594 | LF169 | BM378049 | 2p12 | 2p12 |
| 704 | 585311 | NDL.002K24 | CC851285 | CC851284 | LF409 | BM005511 | 2p34 | 2p34 |
| 710 | 643802 | NDL.090O23 | CC860959 | CC860952 | LF198 | T58319 | 1p32 | 1p32 |
| 766 | 506593 | NDL.005F19 | CC129101 | CC135541 | LF103 | BM005488 | 3q11 | 3q11 |
| 777 | 545594 | NDL.088D2 | NA | NA | LF150 | BM005476 | 1p33 | 1p33 |
| 786 | 488859 | NDL.038M5 | CC151300 | CC151177 | LF291 | BM005482 | 2p42 | 2p42 |
| 816 | 517624 | NDL.079D19 | CC140790 | CC140786 | LF129 | BM005504 | 2q33 | 2q33 |
| 817 | 451076 | NDL.033H12 | NA | CC858631 | *TrypB* | *M77814* | 2p33 | 2p33 |
| 836 | 436213 | NA |  |  | rDNA 18S |  | 1q22 | 1q22 |
| 875 | 466421 | NDL.013I3 | CC136854 | CC136849 | *RpL17A* (LF355) | AF315597 | 2q31*, multiple signals | 2q31 |
| 901 | 389223 | NDL.047B12 | NA | NA | LF261 | BM378052 | 3q23 | 3q23 |
| 1051 | 322300 | NDL.109E9 | CC866919 | CC861722 | *nAcBP* | *AY040341* | 1q42 | 1q42 |
| 1132 | 217800 | NDL.046O19 | CC135531 | NA | BA67 | AI561370 | 2q44 | 2q44 |
| 1168 | 191961 | NDL.040I24 | CC150774 | CC150652 | LF158 | BM005485 | 2p11 | 2p11 |
| 2392 | 11149 | NDL.013I5 | CC137501 | CC137496 | *Tsf* | *AF019117* | 1p33 | 1p33 |
