# Supplementary figures and images for: Genomic composition and evolution of Aedes aegypti chromosomes revealed by the analysis of physically mapped supercontigs
Source: BMC Biol. 2014 Apr 14;12:27. doi: 10.1186/1741-7007-12-27 (PMC4021624; doi:10.1186/1741-7007-12-27)

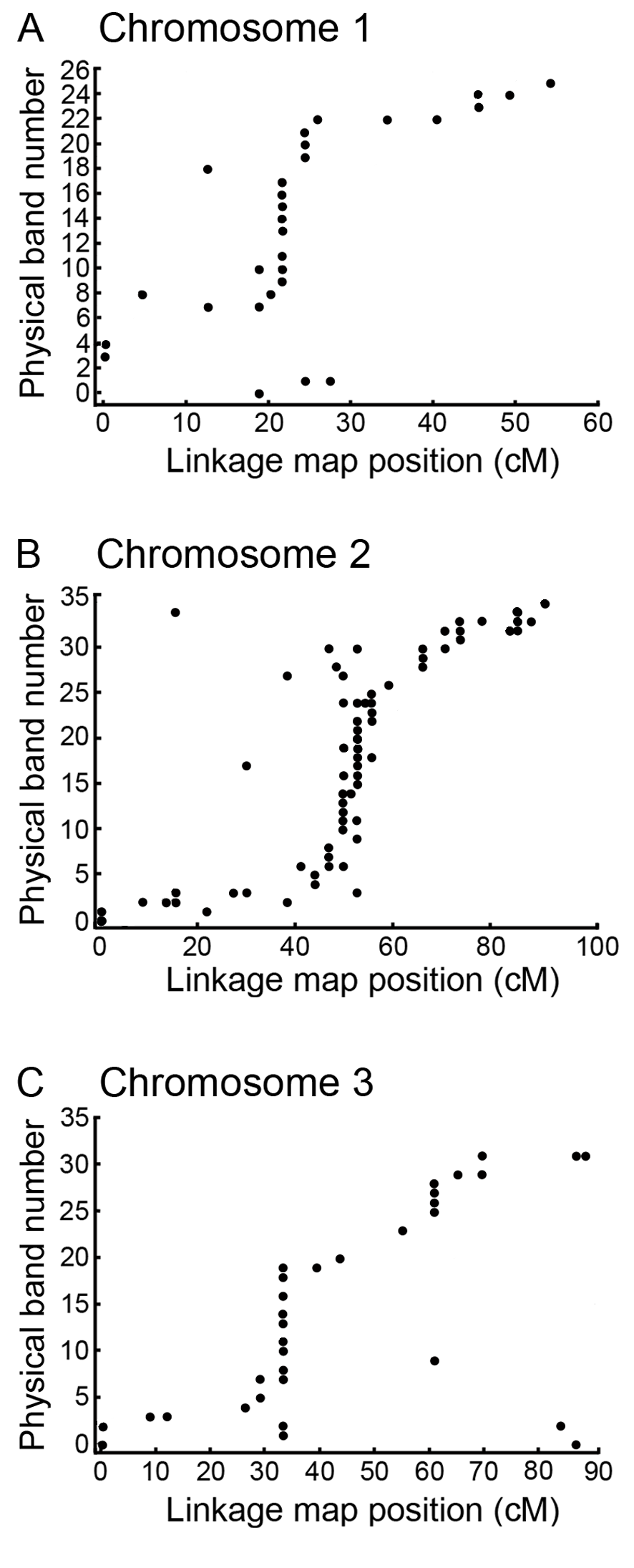

Supplement: Additional file 2: Figure S1 — The correlation between the physical band position of a supercontig on our physical map and its cM position on the genetic linkage map [19]. Spearman’s rank correlation coefficients equal to 0.77, 0.83 and 0.65 (P <0.05) were determined for chromosomes (A) 1, (B) 2 and (C) 3, respectively. [file 1741-7007-12-27-S2.tiff]
